# Chitosan/Hyaluronate Complex-Coated Electrospun Poly(3-hydroxybutyrate) Materials Containing Extracts from *Melissa officinalis* and/or *Hypericum perforatum* with Various Biological Activities: Antioxidant, Antibacterial and In Vitro Anticancer Effects

**DOI:** 10.3390/polym16152105

**Published:** 2024-07-24

**Authors:** Ina Anastasova, Milena Ignatova, Nevena Manolova, Iliya Rashkov, Nadya Markova, Reneta Toshkova, Ani Georgieva, Mariana Kamenova-Nacheva, Antoaneta Trendafilova, Viktoria Ivanova, Tsvetelina Doncheva

**Affiliations:** 1 Laboratory of Bioactive Polymers, Institute of Polymers, Bulgarian Academy of Sciences, Acad. G. Bonchev St., Bl. 103A, BG-1113 Sofia, Bulgaria; i_anastasova@polymer.bas.bg (I.A.); manolova@polymer.bas.bg (N.M.); rashkov@polymer.bas.bg (I.R.); 2 Institute of Microbiology, Bulgarian Academy of Sciences, Acad. G. Bonchev St., Bl. 26, BG-1113 Sofia, Bulgaria; markn@bas.bg; 3 Institute of Experimental Morphology, Pathology and Anthropology with Museum, Bulgarian Academy of Sciences, Acad. G. Bonchev St., Bl. 25, BG-1113 Sofia, Bulgaria; rtoshkova@bas.bg (R.T.); ageorgieva@bas.bg (A.G.); 4 Laboratory for Extraction of Natural Products and Synthesis of Bioactive Compounds, Research and Development and Innovation Consortium, Sofia Tech Park JSC, 111 Tsarigradsko Shose blvd., BG-1784 Sofia, Bulgaria; mariana.nacheva@orgchm.bas.bg; 5 Institute of Organic Chemistry with Centre of Phytochemistry, Bulgarian Academy of Sciences, Acad. G. Bonchev St., Bl. 9, BG-1113 Sofia, Bulgaria; antoaneta.trendafilova@orgchm.bas.bg (A.T.); viktoria.genova@orgchm.bas.bg (V.I.); tsvetelina.doncheva@orgchm.bas.bg (T.D.)

**Keywords:** chitosan, hyaluronate, poly(3-hydroxybutyrate), polyelectrolyte complex, electrospinning, *Melissa officinalis* L. extract, *Hypericum perforatum* extract, antioxidant, antibacterial, anticancer activities

## Abstract

The present study aimed to fabricate innovative fibrous materials with various biological activities from poly(3-hydroxybutyrate), sodium hyaluronate (HA), chitosan (Ch), *Melissa officinalis* (MO), *Hypericum perforatum* (HP) extract, or a combination of both extracts. Electrospinning or electrospinning followed by dip coating and the subsequent formation of a polyelectrolyte complex were the methods used to prepare these materials. Scanning electron microscopy (SEM), transmission electron microscopy (TEM), thermogravimetric analysis (TGA), and attenuated total reflection–Fourier transform infrared spectroscopy (ATR–FTIR) were applied for investigating the morphology of materials, their thermal characteristics, and their surface chemical composition. The composition and design of the mats had an influence on the in vitro release behavior of the main bioactive compounds present in the MO and HP extracts incorporated in the materials. It was found that as-created materials comprising a combination of both extracts and a Ch/HA complex exerted higher antioxidant activity than that of (non-)coated MO-containing mats and Ch/HA-coated mats containing HP. The novel materials manifested antibacterial efficacy towards the pathogenic bacteria *S. aureus* and *E. coli*, as evidenced by the performed microbiological screening. Furthermore, the mats possessed a great growth inhibitory effect on HeLa cancer cells but had a less pronounced effect on the growth of normal mouse BALB/3T3 fibroblasts. The loading of both extracts in the mats and the formation of coating led to the enhancement of the in vitro anticancer and antibacterial activities of the materials. Thus, the novel materials have potential for use in local cancer therapy as well as for use as wound dressings.

## 1. Introduction

Electrospinning is an easily feasible and cost-effective technique for the fabrication of micro- and nanofibrous materials from natural and synthetic polymers [1,2]. Materials obtained by electrospinning having unique features, such as high specific surface area and porosity, can be applicants in a variety of biomedical uses, including wound dressings, tissue engineering scaffolds, biosensors, systems for controlled release of bioactive compounds, hemostatic devices, etc. [2,3,4,5,6]. By means of the electrospinning method, it is possible to easily incorporate low-molecular weight bioactive compounds of various nature into the fibers to improve the biological performance of these materials. Another important asset of electrospinning is the possibility of tuning the release profile of the incorporated compounds. As a result, an enhancement in the therapeutic efficacy of these compounds as well as a reduction in their side effects might be achieved. Electrospun materials are promising carriers of bioactive compounds, which can ensure enhanced stability and bioavailability.

Poly(3-hydroxybutyrate) (PHB), a member of the poly(3-hydroxyalkanoate) family, is suitable for biomedical applications, e.g., as a carrier of bioactive compounds, because of its useful properties such as renewability, biodegradability, biocompatibility, thermoplasticity, non-toxicity, and UV resistance [7,8]. It possesses physical properties similar to polypropylene. In addition, obtaining fibrous materials based on PHB using electrospinning is easily achievable [9,10]. Other bio-based polymers of interest for incorporation into electrospun materials intended for biomedical purposes are polysaccharides, including sodium hyaluronate (HA) and chitosan (Ch). HA is a linear polysaccharide composed of β-(1,4)-D-glucuronic acid and β-(1,3)-N-acetyl-D-glucosamine repeating units, which is a major component of the extracellular matrix that is distributed broadly in the loose connective tissue [11]. It possesses high viscoelasticity, high biocompatibility, non-toxicity, non-immunogenicity, high hydrophilicity, and high capability for moisture retention [12]. HA manifests numerous pharmacological properties, such as wound healing and tissue regenerating, anti-inflammatory, anticancer, anti-diabetic, antioxidant, skin repairing properties, etc. [13,14]. Ch is a linear polysaccharide that is fabricated by partial alkaline N-deacetylation of the natural polymer chitin. It is reported to display a broad range of beneficial properties, such as biodegradability, absence of toxicity, and hemostatic, antibacterial, antioxidant, and anticancer properties [15,16,17]. In aqueous solutions, chitosan has the capability to form polyelectrolyte complexes (PECs) with polyanions [15,18,19]. In recent years, Ch/HA PECs have been obtained in order to create materials prospective for biomedical uses, such as systems for drug delivery, wound healing, cell proliferation, etc. [18,19].

Plant extracts are particularly attractive for use in medicine, pharmacy, cosmetics and as functional foods due to the valuable biological properties (antimicrobial, antifungal, anti-inflammatory, antioxidant, and anticancer activities) they possess [20,21,22]. The incorporation of these extracts into fibrous materials prepared by electrospinning can impart desirable biological activity to them. Different electrospinning techniques and approaches can be applied for the fabrication of plant extract-containing fibrous materials of various design: blend electrospinning, emulsion electrospinning, co-axial electrospinning, dual spinneret electrospinning, and electrospinning in conjunction with other techniques (electrospraying, dip coating). In order to prepare these materials, it is necessary to achieve control over the parameters of the spinning solution and the electrospinning process. The loading of plant extracts to the spinning solution may influence the spinnability of the solution, which is important for defining the morphology, composition, and properties of the materials.

*Melissa officinalis* L. (MO) is a plant belonging to the *Lamiaceae* family used in traditional medicine. Some of the biological activities of this medicinal plant are associated with the phenolic acids and flavonoids found in MO extracts. The extracts of MO have several activities—antibacterial [23], antifungal [24], anticancer [25], antioxidant [26], antiviral [27], antiangiogenic [28], antidepressant [29], anti-Alzheimer [30], neuroprotective [31], and cardioprotective effects [32]. Furthermore, extracts from the plant *Hypericum perforatum* (HP), St. John’s wort, a member of the *Hypericaceae* family, have long been regarded as an effective natural remedy. HP extracts are known for their use in the treatment of depression [33]. In addition, extracts from this plant possess antibacterial [34], antifungal [35], anticancer [36], antioxidant [37], anti-inflammatory [38], antiviral [39], and wound-healing [40] activities, etc., which is attributed to the presence of secondary metabolites such as naphthodianthrones (hypericin and its derivatives), phloroglucinols (hyperforin), flavonoids (biapigenin, quercetin, and its glycosides), phenolic acids (chlorogenic acid), etc.

So far, there is a limited number of investigations on the preparation of electrospun materials as carriers of essential oils or extracts of MO. Recently, some of us have shown that the incorporation of MO extract into polylactide or polylactide/polyethylene glycol fibrous materials by electrospinning imparts high antioxidant activity to the materials [41]. Encapsulation of lemon balm (MO) and dill (*Anethum graveolens* L.) essential oils in collagen hydrolysate/chitosan fibrous materials by coaxial electrospinning was shown to result in materials with improved antimicrobial activity [42]. Moreover, fibrous materials loaded with HP extract or HP-containing nanoliposomes have been prepared by one-pot electrospinning from synthetic polymers (polyvinyl alcohol [43], thermoplastic polyurethane [43]) or bio-based polymers (polylactic-*co*-glycolic acid [44], poly(ε-caprolactone) [43,45], polylactic acid [43], cellulose acetate [43], Ch [43], hydrolyzed collagen/polyvinyl alcohol [46], fungal Ch/polycaprolactone [47]). Double-layer electrospun fibrous materials, composed of polylactic acid as an upper layer and a blend of polyethylene oxide and Ch containing HP as a lower layer, designed to be in contact with the wound have been prepared by electrospinning and electrospraying [48]. PHB-based fibrous mats containing hypericin-rich HP extract have been obtained by centrifugal spinning technology [49]. Mouro et al. [50] have fabricated electrospun HP-loaded poly(L-lactic acid)/poly(vinyl alcohol)/Ch fibrous materials by emulsion electrospinning. All of these fibrous materials containing HP possess good antibacterial activity. However, their anticancer activity has not been evaluated.

As far as we are aware, there are no data in the literature related the preparation of MO-loaded PHB fibrous materials coated with PEC Ch/HA (non-)containing HP extract. In the present study, the extracts of MO and HP have been chosen because of their biological properties, such as their antioxidant, antibacterial, and anticancer effects. It can be assumed that the use of these properties of the extracts and the useful features of the natural polysaccharides Ch and HA and polyester PHB will impart a beneficial biological activity to the prepared materials.

In the present contribution, our objective was to investigate the possibility for fabrication of novel fibrous materials of diverse designs based on PHB, Ch, and HA containing MO, HP, or a combination of both plant extracts by using one-pot electrospinning or electrospinning followed by dip coating and subsequent PEC formation. The novel materials were characterized with respect to the morphology, wettability, crystallinity, and thermal characteristics. An evaluation of their efficacy against the pathogenic bacteria *S. aureus* and *E. coli* was performed. The effect of the composition of the prepared materials on their antioxidant activity and in vitro anticancer properties towards HeLa cancer cells was assessed as well.

## 2. Materials and Methods

### 2.1. Materials

PHB with ${\bar{M}}_{n}$ 330,000 g·mol^−1^ (Biomer, Schwalbach am Taunus, Germany) and sodium hyaluronate (HA) (Acros Organics, Geel, Belgium) were used. Chitosan (Ch) with average viscometric molar mass 380,000 g·mol^−1^ and deacetylation degree 80% was acquired from Aldrich (St. Louis, MO, USA). Dimethylformamide (DMF) (Merck, Billerica, MA, USA), chloroform (Merck, Billerica, MA, USA), glacial acetic acid (Merck, Billerica, MA, USA), and absolute ethanol (Merck, Billerica, MA, USA) were of analytical grade purity. Chlorogenic acid (5-CQA), rutin, isoquercitrin (quercetin 3-*O*-glucoside), quercetin, and hypericin was supplied by Phytolab GmbH & Co. KG, Vestenbergsgreuth, Germany. All solvents used for HPLC and HPLC-ESI/MS were of HPLC and LC-MS grade.

The “Essential Oils and Herbs” Company Ltd. supplied plant material from cultivated MO (grown in Blatets Village, Bulgaria). The aerial parts of HP were purchased from Dicrassin Bulgaria Ltd. (Sofia, Bulgaria).

*S. aureus* strain 749 and *E. coli* strain 3588 were acquired from the National Bank for Industrial Microorganisms and Cell Cultures, Sofia, Bulgaria.

Human cervical adenocarcinoma (HeLa) cancer cells and mouse BALB/3T3 clone A31 cells were obtained from the American Type Culture Collection (ATCC, Manassas, VA, USA). Sigma-Aldrich, Schnelldorf, Germany, supplied acridine orange (AO), ethidium bromide (EtBr), 3-(4,5-Dimethylthiazol-2-yl)-2,5-diphenyltetrazolium bromide (MTT), and Dulbecco’s modified Eagle medium (DMEM). The cell culture reagents fetal bovine serum (FBS) (Gibso/BRL, Grand Island, NY, USA), L-glutamine, penicillin, and streptomycin solution (LONZA, Cologne, Germany) were used. Moreover, AppliChem, Darmstadt, Germany, delivered the 4′,6-diamidino-2-phenylindole (DAPI). The plastic consumables were acquired from Orange Scientific, Braine-l’Alleud, Belgium.

### 2.2. Extraction of Plant Material

#### 2.2.1. Extraction of MO

The procedure for obtaining the MO dry extract used in these studies was previously reported [41]. The aerial parts of the MO species were extracted with 70% aqueous methanol for 24 h at room temperature. The extract was filtered, and the solvent was evaporated under reduced pressure to produce MO dry extract.

#### 2.2.2. Extraction of HP

The aboveground parts of *H. perforatum* (HP, 100 g) were extracted with 60% aqueous ethanol for 5 h at 50 °C. HP dry extract (20.7 g) was obtained after removing the solvent and concentration by a rotary vacuum evaporator.

### 2.3. Characterization and Quantification of Dry Extracts of MO and HP by HPLC-DAD-ESI/MS

#### 2.3.1. Characterization of Dry Extracts of MO and HP by HPLC-DAD-ESI/MS

The identification of the main components in MO dry extract was performed using HPLC-DAD-ESI/MS analysis on Shimadzu LC-2040C 3D Nexera-i and Shimadzu LCMS 2020 (single quadrupole) systems (Tokyo, Japan). The chromatographic and mass spectrometric conditions were as given in reference [41].

The chemical characterization of HP dry extract was carried out on the same equipment as in reference [41]. The solvents used for the analysis were (A) NH_4_OAc (10 mM, pH 4.56), (B) CH_3_CN, and (C) CH_3_OH, as described in reference [51]. The gradient of chromatographic conditions is given in Table S1 (Supplementary Materials).

#### 2.3.2. Quantification of Rosmarinic Acid in MO Extract

The quantification of rosmarinic acid in MO is described in the Supplementary Materials (see Quantification of Rosmarinic Acid in MO extract).

#### 2.3.3. Quantification of the Compounds in HP Extract

The quantification of the compounds in HP extract was conducted using HPLC-DAD analysis on a Shimadzu Nexera-i LC-2040C 3D Plus liquid chromatograph equipped with a photodiode array detector (Shimadzu, Tokyo, Japan) at the conditions described in Section 2.3.1. Different wavelengths were used for the monitoring of phloroglucinols (290 nm), phenolic acids (320 nm), flavonoids (360 nm), and hypericins (590 nm). Chlorogenic acid (0.009–0.152 mg/mL, R^2^—0.9999), rutin (0.014–0.218 mg/mL, R^2^—0.9999), isoquercitrin (0.006–0.100 mg/mL, R^2^—1.0), quercetin (0.003–0.046 mg/mL, R^2^—1.0), I3,II8-biapigenin (0.007–0.112 mg/mL, R^2^—0.9999), hypericin (0.002–0.040, R^2^—0.9993), and hyperforin (0.006–0.100, R^2^—0.9990) in different concentrations were used as standards. Neochlorogenic acid (3-CQA) was calculated as equivalents of chlorogenic acid (5-CQA). The quantities of hyperoside, miquelianin, quercitrin, and quercetin 3-O-(O-acetyl-hexoside) were expressed as equivalents of isoquercitrin. The quantity of protopseudohypericin, pseudohypericin, and protohypericin was determined as equivalents of hypericin and that of adhyperforin as hyperforin equivalents. All experiments were performed in triplicate. The contents were expressed in mg/g DE (dry extract). In the in vitro release study, the injected volume was 10 μL of each sample, and the total flavonoids and hypericins were determined as a sum of the amount of all identified flavonoids and naphthodianthrones, respectively, expressed in mg/g DE.

#### 2.3.4. Isolation of I3,II-biapigenin and Hyperforin

The isolation of I3,II8-biapigenin and hyperforin is described in the Supplementary Materials (see Isolation of I3,II8-biapigenin and Hyperforin). Their structures were confirmed by ^1^H NMR spectroscopy (Supplementary Materials, Figures S1–S3) and compared with the literature data [52,53].

### 2.4. Preparation of the Fibrous Mats

The composition of fabricated fibrous mats are given in Table 1.

#### 2.4.1. Preparation of Neat and MO-Containing PHB Mats by Electrospinning

The PHB mats were obtained by electrospinning its solution in CHCl_3_/DMF 4/1 (*v*/*v*) at a concentration of 10 wt%. MO-containing PHB (MO-*in*-PHB) mats were fabricated from solutions prepared after mixing a solution containing PHB (6.0 g) dissolved in 33 mL of CHCl_3_ under heating at 60 °C and a solution containing different amounts of dry MO (5 and 10 wt% with respect to the polymer weight) dissolved in 8.1 mL of DMF. The electrospinning of PHB and MO-*in*-PHB solutions was conducted as follows: rate of delivering of the solution of 2 mL/h (infusion pump: Syringe Pump NE-300 (New Era Pump Systems, Inc., Farmingdale, NY, USA); voltage of 26 kV (high-voltage power supply (10–30 kV)); tip-to-collector distance of 25 cm; and use of a grounded rotating aluminum drum collector (rotating rate of 1300 rpm).

#### 2.4.2. Preparation of Ch/HA-*coat*-PHB Mats by Electrospinning, Dip Coating, and PEC Formation

An amount of 0.05 g of HA was dissolved in 50 mL of acetate buffer (pH 4.5) at 25 °C overnight. Then, Ch was dissolved in diluted acetic acid (1% *v*/*v*) at a concentration of 0.1 wt% under continuous stirring for 24 h at room temperature. Then, the pH of the Ch solution was adjusted to 4.5 with 0.1 M sodium hydroxide. For the preparation of PHB mats coated with HA (designated as HA-*coat*-PHB mats), PHB mats were soaked in a 0.1 wt% solution of HA in acetate buffer (pH 4.5) for 10 min and dried at 25 °C for 1 day. Then, the HA-*coat*-PHB mats was immersed into a 0.1 wt% solution of Ch for 10 min, rinsed with distilled water, and dried to a constant weight.

#### 2.4.3. Preparation of (Ch,HP)/HA-*coat*-PHB and (Ch,HP)/HA-*coat*-(MO-in-PHB) Mats by Electrospinning, Dip Coating, and PEC Formation

First, a 0.2 wt% Ch solution was obtained in diluted acetic acid (1% *v*/*v*) under stirring for 24 h. The pH of the Ch solution was adjusted to 4.5 with 0.1 M sodium hydroxide. Then, 0.02 g of HP was dissolved in 5 mL of ethanol/distilled water = 3/2 (*v*/*v*). The HP solution was added dropwise to the above-prepared Ch solution (5 mL) by stirring for 4 h. For the preparation of PHB mat coated with HP-containing Ch/HA (denoted as (Ch,HP)/HA-*coat*-PHB), HA-*coat*-PHB mats were immersed into the Ch solution containing HP, left in the solution for 10 min, withdrawn from the solution, washed with distilled water, and dried to a constant weight. The amount of the incorporated HP was 20 wt% with respect to the PHB weight and was determined spectrophotometrically after 24 h stay under stirring of the respective mats in ethanol/distilled water = 3/2 (*v*/*v*) (λmax 587 nm, ethanol/water = 3/2 (*v*/*v*)).

Coating of (Ch,HP)/HA on the MO-*in*-PHB mat surface was obtained by immersing the HA-coated mats into the above-prepared Ch solution containing HP at room temperature for 10 min. The mats were washed with distilled water and dried to a constant weight. The content of the incorporated HP was 20 wt% relative to the PHB weight and was determined spectrophotometrically after a 24 h stay under stirring of the respective mats in ethanol/distilled water = 3/2 (*v*/*v*) (λmax 587 nm, ethanol/water = 3/2 (*v*/*v*)).

For the preparation of PEC-coated PHB and MO-*in*-PHB mats with HP content 10 wt% relative to the PHB weight (determined spectrophotometrically (λmax 587 nm, ethanol/water = 3/2 (*v*/*v*)), the HA-coated mats were placed in a mixture of 5 mL of 0.2 wt% Ch solution in diluted acetic acid (1% *v*/*v*), whose pH was adjusted to 4.5, and 5 mL of 0.2 wt% HP solution in ethanol/water (3/2, *v*/*v*) at 25 °C for 10 min, and they were washed with distilled water and then dried to constant weight.

### 2.5. Fibrous Mats Characterization

Scanning electron microscopy (SEM, Jeol JSM-5510, Tokyo, Japan) was utilized to observe the morphology of the obtained fibrous materials after gold coating (Jeol JFC-1200 fine coater). ImageJ software (V.1.53 e, Wayne Rasband, National Institute of Health, Bethesda, MD, USA) was applied to calculate the mean diameter of fibers and standard deviation by measuring the diameters of at least 100 fibers. TEM analyses were carried out by JEM 2100 (JEOL Ltd., Tokyo, Japan) operating at 200 kV. A Bookfield DV-II+ viscometer delivered by Middleboro, MA, USA, with a thermostatic cup and a cone spindle was applied to measure the dynamic viscosity of solutions at room temperature (25 °C).

Attenuated total reflection–Fourier transform infrared (ATR–FTIR) spectra were acquired by an IRAffinity-1 spectrophotometer supplied by Shimadzu Co., Kyoto, Japan. It was fitted with a MIRacleTM ATR accessory with a diamond crystal (PIKE Technologies, Madison, WI, USA). The spectra were recorded from 600 to 4000 cm^−1^.

Thermogravimetric analysis (TGA) was conducted on a Perkin Elmer TGA 4000 produced from Waltham, MA, USA, in an argon environment. The heating of the samples was from room temperature to 800 °C at a heating rate of 10 °C/min. Pyris v.11.0.0.0449 software was applied to control, collect, and process data from the instrument.

X-ray diffraction analyses (XRDs) of the plant extracts and fibrous mats were carried out at room temperature on a D8 Bruker Advance powder diffractometer (Billerica, MA, USA) filtered with Cu Kα radiation. The specimens were step-scanned from 5 to 60° in 2*θ* with steps of 0.02° with a counting time of 1 s/step.

The hydrophobic/hydrophilic behavior of the mat surface was analyzed by performing the static contact angle measurements on an Easy Drop drop shape analysis system DSA20E (Krűss GmbH, Hamburg, Germany) at 20 ± 0.2 °C. Drops of distilled water (10 L) were placed on the mat surface. Computer analysis of the droplet’s temporal images was utilized to determine the water contact angles. The mean contact angle value is the average of at least 20 measurements performed on different areas of the mat surfaces.

To determine the weight losses of Ch/HA-*coat*-PHB mats in PBS (pH 7.4), the mats were soaked in PBS for 24 h. The treated mats were rinsed with distilled water and freeze-dried. The weight losses of the treated mats were determined gravimetrically.

### 2.6. In Vitro Release Studies

The in vitro release of rosmarinic acid, one of the major bioactive compounds found in MO extract, as well as the release of neochlorogenic acid, total flavonoids, and total hypericins, the main bioactive compounds found in HP extract, from the prepared fibrous materials containing MO, HP, or both extracts were conducted in PBS solution(pH 7.4) with an ionic strength of 0.1 at 37 °C. The fibrous mats (19 mg) were placed in 30 mL of PBS and were thermostated in a shaker bath ((JULABO SW23, Allentown, PA, USA) at 37 °C under constant stirring at 100 rpm. At specific time points, aliquots (2 mL) were extracted from the solution and replenished with fresh PBS.

The amount of the released rosmarinic acid, neochlorogenic acid, total flavonoids, and total hypericins was evaluated by HPLC-DAD-ESI/MS analysis, as described in Section 2.3. The cumulative percentage of the released rosmarinic acid, neochlorogenic acid, total flavonoids, and total hypericins was calculated as a function of incubation time. The averaged data from three measurements are presented.

### 2.7. Antioxidant Activity of the Mats

The antioxidant activities of the plant extracts and prepared mats were determined by a DPPH free radical scavenging test. A total of 0.0033 g of MO-*in*-PHB and 0.0039 g of (Ch,HP)/HA-*coat*-PHB, (Ch,HP)/HA-*coat*-(MO-*in*-PHB), and Ch/HA-*coat*-PHB mats were immersed in 0.5 mL of ethanol/distilled water (7/3, *v*/*v*), and then 1.5 mL of DPPH solution in ethanol (0.1 mM) was added. A 0.5 mL solution of MO or of HP containing 0.3 mg of MO (HP) in ethanol/distilled water (7/3, *v*/*v*) was added to 1.5 mL of 0.1 mM solution of DPPH in ethanol. The solutions were left in the dark for 30 min. After this period, absorbances were recorded by a DU 800 UV-Vis spectrophotometer acquired by Beckman Coulter, Brea, CA, USA, at 517 nm. The DPPH scavenging effect was measured as follows:

Inhibition, AA, % = [(A_DPPH●_ − A_sample_)/A_DPPH●_] × 100 (1)

where AA,% represents the antioxidant activity. A_DPPH●_ is the absorbance of DPPH• at 517 nm; A_sample_ is the absorbance of DPPH• solution with tested sample at 517 nm. Each sample was tested three times.

### 2.8. Microbiological Tests against S. aureus and E. coli

The antibacterial activity of the prepared fibrous mats was determined in vitro against the pathogenic bacteria *S. aureus* 749 and *E. coli* 3588 by the disk diffusion method using tryptic soy agar (TSA) solid medium supplied by Becton Dickinson, Heidelberg, Germany. The Petri dishes with solid agar were inoculated with a suspension of *S. aureus* or *E. coli* (concentration of 1 × 10^5^ cells/mL). The discs from fibrous mats (diameter of 19 mm) were placed on the inoculated surface of the agar. The dishes were incubated for 24 h at 37 °C. Furthermore, the mean diameters of the sterile zones formed around each disk were determined by ImageJ software (V.1.53 e,Wayne Rasband, National Institute of Health, Bethesda, MD, USA) based on 20 measurements.

### 2.9. Evaluation of the In Vitro Anticancer Activity

#### 2.9.1. Cell Culturing

HeLa and BALB/3T3 cells were grown in 25 cm^2^ cell culture flasks in DMEM medium supplemented with 10% fetal bovine serum, 100 U/mL penicillin, and 100 μg/mL streptomycin in a CO_2_ incubator (Thermo Scientific, Waltham, MA, USA, HEPA Class 100) at 37 °C and humidified atmosphere with 5% CO_2_. A solution containing 0.05% trypsin and 0.02% EDTA was used for cell detachment.

#### 2.9.2. Cytotoxicity Assay

The cytotoxicity of the prepared fibrous materials on human HeLa cancer cells and BALB/3T3 non-cancerous mouse fibroblasts was determined using the MTT proliferation assay. Briefly, cancer cells were seeded in 96-well plates in DMEM medium at a density of 1 × 10^4^ cells per well. After an incubation period of 24 h at 37 °C and 5% CO_2_, the cells were incubated with fresh medium alone or in the presence of various fibrous materials (PHB, Ch/HA-*coat*-PHB, MO-*in*-PHB, (Ch,HP)/HA-*coat*-PHB, (Ch,HP)/HA-*coat*-(MO-*in*-PHB)); and solutions of MO, HP, and a mixture of both extracts (MO:HP extracts (1:2 *w*/*w*)) for 72 h. The effects of MO and HP on HeLa cell viability were compared with those of the standard chemotherapeutic agent doxorubicin (DOX). All MO- and HP-containing fibrous mats were investigated at an MO and HP concentration of 130 μg/mL and 260 μg/mL of culture medium, respectively. The concentrations of MO and HP in the studied solutions were 130 μg/mL and 260 μg/mL, respectively. Untreated cells cultures were used as a control. At 24 h and 72 h of incubation, the studied formulations (mats or solutions) and culture medium were removed, and MTT solution (0.5 mg mL^−1^; 100 µL per well) and the cells were re-incubated for 3 h. At the end of the incubation, the MTT-containing medium was removed, and solubilizing solution (DMSO/EtOH, 1:1, *v*/*v*) was added to dissolve formazan crystals. Finally, the absorbance was read by an ELISA plate reader (TECAN, Sunrise^TM^, Grödig/Salzburg, Austria) at a wavelength of 570 nm. The cell viability was presented as a percentage of the negative control cells.

All experiments were performed in six replicates.

#### 2.9.3. Cytomorphological Fluorescence Microscopy Analyses

To analyze the mechanisms underlying the cytotoxic effects of the studied formulations (fibrous materials and solutions), the cytomorphological alterations induced in the cancer and non-cancerous cells were examined by fluorescent microscopy. The HeLa and BALB/3T3 cells were plated at a density of 2 × 10^5^/mL on glass coverslips placed into 24-well plates and incubated for 24 h in an incubator at 37 °C and 5% CO_2_. The cell cultures were then exposed to the tested fibrous mats (PHB, Ch/HA-*coat*-PHB, MO-*in*-PHB, (Ch,HP)/HA-*coat*-PHB, (Ch,HP)/HA-*coat*-(MO-*in*-PHB)) and solutions of MO, HP, and a mixture of both extracts (MO:HP extracts (1:2 *w*/*w*)) for 24 h. At the end of the treatment, the glass coverslips were rinsed with PBS, and two fluorescent staining methods were applied. Vital staining with AO/EtBr was used to discriminate between dead and viable cells in the treated cell cultures and to identify apoptotic morphology changes. The fluorescent dyes were dissolved in PBS at a concentration of 10 μg/mL and applied to the native cell preparations. For more detailed investigation of the nuclear morphology changes, staining with the DNA-binding stain DAPI was performed. The cell preparations were fixed and stained with a 10 μg/mL methanol solution of DAPI for 10 min in the dark at room temperature and then rinsed with methanol and mounted on microscope slides. The fluorescently stained cell cultures were visualized using a fluorescent microscope (Leica DM 5000B, Wetzlar, Germany).

### 2.10. Statistical Analysis

The cell viability data are presented as the mean ± standard deviation (SD), and a one-way analysis of variance (ANOVA) was utilized to assess the significance of the differences between the control and treated cell cultures using GraphPad PRISM (Version 5). Values of *p* < 0.05 were accepted as the lowest level of statistical significance. A nonlinear regression curve fit analysis (GraphPad PRISM) was applied to determine the 50% inhibitory concentrations (IC_50_).

## 3. Results and Discussion

### 3.1. Phytochemical Characterization of MO and HP Dry Extracts by HPLC-DAD-ESI/MS

#### 3.1.1. Phytochemical Characterization of MO Dry Extract

In a previous study of MO dry extract by HPLC-DAD-ESI/MS [41], it was found that MO extract was rich in phenolic acids such as rosmarinic, caffeic, caftaric, sagerinic, sulphated rosmarinic, salvianolic, etc., acids, while luteolin 7-O-glucuronide was the only flavonoid detected in MO. Among them, rosmarinic acid was the major compound, whose content as determined by HPLC-DAD was found to be 76.27 ± 0.1 mg/g. The presence of this compound had an impact on the biological activity displayed by MO extracts.

#### 3.1.2. Phytochemical Characterization of HP Extract

The HPLC-DAD-ESIMS analysis of HP extract in the negative ionization mode led to the identification of 18 compounds (Supplementary Materials, Table S2, Figures S4 and S5) by a comparison of their spectral characteristics with those of standards or previously published data (for phytochemical characterization of HP extract, see the Supplementary Materials). Among individual compounds, rutin (45.74 ± 0.80 mg/g), hyperoside (14.17 ± 0.38 mg/g), neochlorogenic acid (11.18 ± 0.60 mg/g), and I3,II8-aiapigenin (10.52 ± 0.29 mg/g) were found in amounts that were higher than 10 mg/g DE (Supplementary Materials, Table S2). The determined content of total hypericins (0.30%), total flavonoids (8.68%), and hyperforin (3.34%) corresponds to the requirements listed in the *European Pharmacopeia* for a standardized St. John’s wort dry extract, i.e., 0.1–0.3% of total hypericins, a minimum 6.0% of total flavonoids, and a maximum 6.0% of hyperforin [54]. The above-described secondary metabolites in HP extracts have been reported to be responsible for various biological properties of the HP extracts.

### 3.2. Preparation and Morphology of the Fibrous Mats

Combining the advantageous properties of the aliphatic polyester PHB and natural polymers Ch and HA with the beneficial biological activities (antioxidant, antibacterial, and anticancer) of extracts of MO and HP is a prospective technique for the development of innovative materials suitable for a wide range of biomedical applications.

**Scheme 1:**
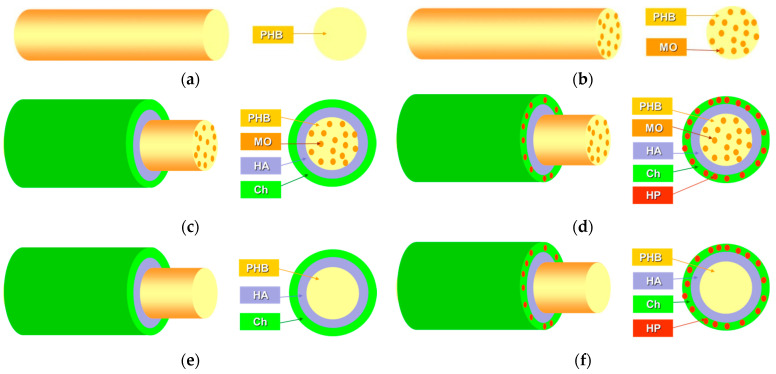
Schematic representation of the developed fibrous materials: (**a**) PHB; (**b**) MO-*in*-PHB; (**c**) Ch/HA-*coat*-(MO-*in*-PHB); (**d**) (Ch,HP)/HA-*coat*-(MO-*in*-PHB); (**e**) Ch/HA-*coat*-PHB; and (**f**) (Ch,HP)/HA-*coat*-PHB.

The fibrous materials with diverse designs that were fabricated in the current work are shown in Scheme 1, namely (i) PHB fibers loaded with MO (MO-*in*-PHB, Scheme 1b), (ii) MO-*in*-PHB fibers coated with Ch/HA PEC (Ch/HA-*coat*-(MO-*in*-PHB), Scheme 1c), and (iii) MO-*in*-PHB fibers coated with Ch/HA PEC containing HP ((Ch,HP)/HA-*coat*-(MO-*in*-PHB), Scheme 1d). PHB (Scheme 1a), PEC-coated PHB mats (not) containing HP (Ch/HA-*coat*-PHB (Scheme 1e), and (Ch,HP)/HA-*coat*-PHB mats (Scheme 1f)) were also obtained.

**Figure 1:**
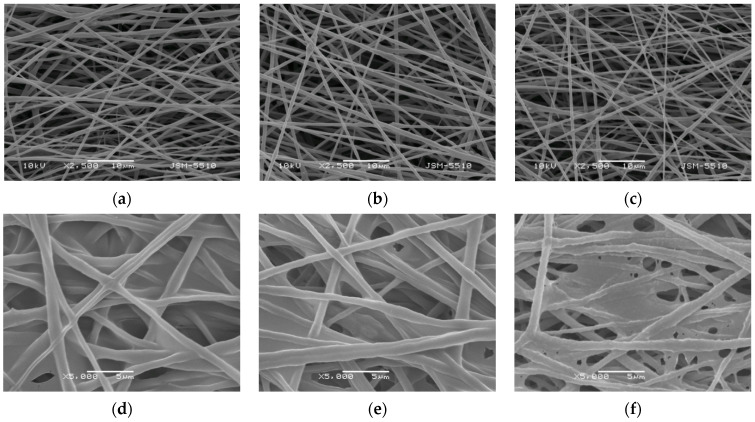

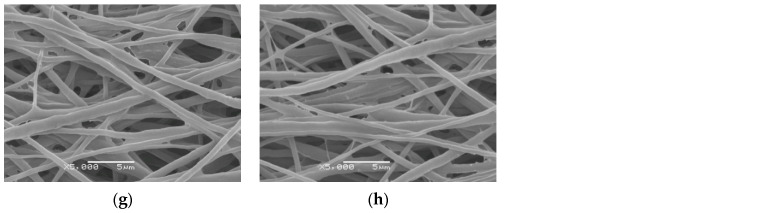
SEM micrographs of the prepared fibrous mats: (**a**) PHB; (**b**) MO-*in*-PHB (5 wt.% MO); (**c**) MO-*in*-PHB (10 wt.% MO); (**d**) Ch/HA-*coat*-PHB; (**e**) (Ch,HP)/HA-*coat*-PHB (20 wt.% HP); (**f**) Ch/HA-*coat*-(MO-*in*-PHB) (10 wt.% MO); (**g**) (Ch,HP)/HA-*coat*-(MO-*in*-PHB) (10 wt.% MO; 10 wt.% HP); and (**h**) (Ch,HP)/HA-*coat*-(MO-*in*-PHB) (10 wt.% MO; 20 wt.% HP). Magnification ×2500 (**a**-**c**) and ×5000 (**d**–**h**); scale bar = 10 µm (**a**–**c**) and 5 µm (**d**–**h**).

The chosen electrospinning conditions (see Section 2.4.1.), polymer concentration of 10 wt.%, and suitable solvent system (CHCl_3_/DMF = 4/1 (*v*/*v*)) were found to be appropriate for producing cylindrical and defect-free PHB fibers containing 5 and 10 wt.% MO extract. The mean diameters of the prepared PHB fibers containing 5 wt% and 10 wt% MO extract were 615 ± 240 nm and 535 ± 160 nm, respectively (Figure 1b,c). Under the same conditions, fibers prepared from a PHB solution had a mean diameter of 730 ± 320 nm (Figure 1a).

The dynamic viscosities of neat PHB and PHB solutions containing 5 wt% and 10 wt% MO were 415, 350, and 270 cP, respectively. It is evident that loading the mixture of low-molecular weight compounds present in the MO extract to the PHB solutions resulted in a slight decline in the viscosity of the spinning solutions. The detected smaller mean diameters of MO-*in*-PHB fibers compared with PHB fibers might be ascribed to the slight reduction in the viscosity of the spinning solutions of PHB in the presence of MO.

The electrospun PHB and MO-*in*-PHB mats were coated with a film of HA followed by the formation of PEC Ch/HA (non-)containing HP. To facilitate the electrostatic interactions between the carboxylate groups of HA and the protonated amino groups of Ch, which allow for the formation of the PEC Ch/HA coating, the pH value of the polyelectrolyte solutions was 4.5. At this pH, the theoretical percentage of ionization of both polyelectrolytes is close to maximum (pKa of HA = 2.9 [55] and pKa of Ch = 6.5 [56]). From the presented SEM micrographs, it is evident (Figure 1d–h) that coating the fibers with a Ch/HA complex and with a Ch/HA complex containing HP led to an increase in the mean fiber diameter. The films between the fibers were also recorded (Figure 1d–h). The values of the mean fiber diameters determined from the SEM micrographs were 790 ± 320 nm, 820 ± 300 nm, and 710 ± 230 nm for the Ch/HA-*coat*-PHB, (Ch,HP)/HA-*coat*-PHB, and Ch/HA-*coat*-(MO-*in*-PHB) mats, respectively. In the case of (Ch,HP)/HA-*coat*-(MO-*in*-PHB) fibrous materials containing 10 wt% and 20 wt% HP, the mean diameters were 700 ± 200 nm and 700 ± 260 nm, respectively.

**Figure 2:**
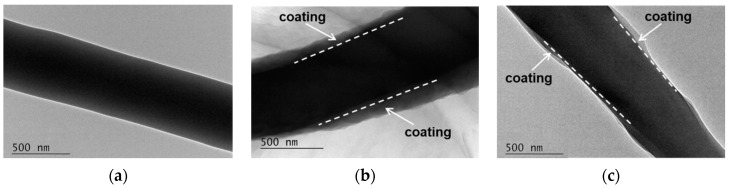
TEM images of a fiber of: (**a**) MO-*in*-PHB mat (10 wt.% MO); (**b**) Ch/HA-*coat*-(MO-*in*-PHB) mat (10 wt.% MO); and (**c**) (Ch,HP)/HA-*coat*-(MO-*in*-PHB) mat (10 wt.% MO; 20 wt.% HP). Magnification: × 10,000.

Additional confirmation of the formation of a coating on the fiber surface was also obtained from the conducted TEM analyses (Figure 2). These analyses clearly demonstrated that unlike the single fiber from the MO-*in*-PHB mat, which had a regular cylindrical shape, a smooth surface and a mean diameter of 600 nm, the same fibers after coating with Ch/HA (not) containing HP had an irregular surface, and due to the difference in densities of PHB and Ch/HA, the PEC coating could be detected as a light outer layer around the dark fiber of MO-*in*-PHB. The mean diameters of coated fibers were around 724 nm and 650 nm for fibers from the Ch/HA-*coat*-(MO-*in*-PHB) and (Ch,HP)/HA-*coat*-(MO-*in*-PHB) mats, respectively.

To investigate the stability of the Ch/HA coating on the surface of PHB mats in a neutral medium, these materials were soaked in PBS (pH 7.4) for 24 h. After that, the mats were rinsed with distilled water and freeze-dried. No weight loss was recorded. Therefore, the Ch/HA coating was sufficiently stable under the described conditions.

### 3.3. Wettability of the Mats

**Figure 3:**
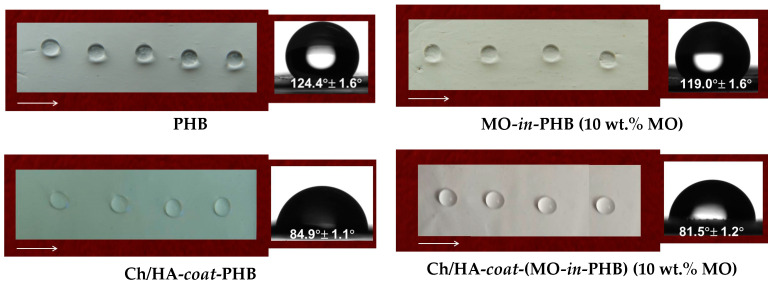

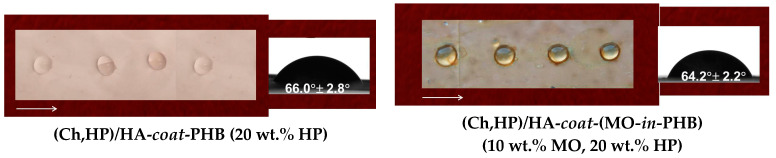
Images of droplets of water placed on the surface of fibrous mats.

It is known that the hydrophilic/hydrophobic properties of surfaces can influence the adhesion of bacterial and cancer cells, their proliferation, and differentiation [57]. Water contact angle (WCA) determination was performed to study the wettability of the (non-)coated mats containing MO, HP, or both extracts. Figure 3 shows the results of the WCA measurements for the investigated fibrous materials. A WCA of 124.4 ± 1.6° was determined for the PHB mat. The incorporation of MO did not affect the WCA values of the mats significantly. The MO-*in*-PHB mats are hydrophobic with values of the WCA being 119.0 ± 1.6°. Coating the PHB and MO-*in*-PHB mats with PEC leads to a decrease in the WCA, reaching values of 84.9 ± 1.1° and 81.5 ± 1.2°, respectively. These lower values indicate an enhancement in the hydrophilicity of the materials. The WCA values of the (Ch,HP)/HA-coated PHB, and MO-*in*-PHB mats (Figure 3) are slightly lower than those of Ch/HA-*coat*-PHB and Ch/HA-*coat*-(MO-in-PHB) mats (Figure 3). This is most likely due to the incorporation of polar hydrophilic compounds present in the HP extract to the surface of the mats. The obtained results are consistent with data from other studies that have demonstrated a drop in the WCA when HP extract was incorporated into electrospun poly(L-lactic acid)/poly(vinyl alcohol)/Ch fibrous mats [50]. These results showed that (Ch,HP)/HA-*coat*-PHB and (Ch,HP)/HA-*coat*-(MO-*in*-PHB) mats had slightly higher wettability compared with mats that do not contain HP. The hydrophilicity is regarded as an important feature in order to accomplish a rapid therapeutic efficacy of the incorporated extracts considering the potential future application of the obtained materials in the biomedical field.

### 3.4. ATR–FTIR Spectroscopy of the Mats

**Figure 4:**
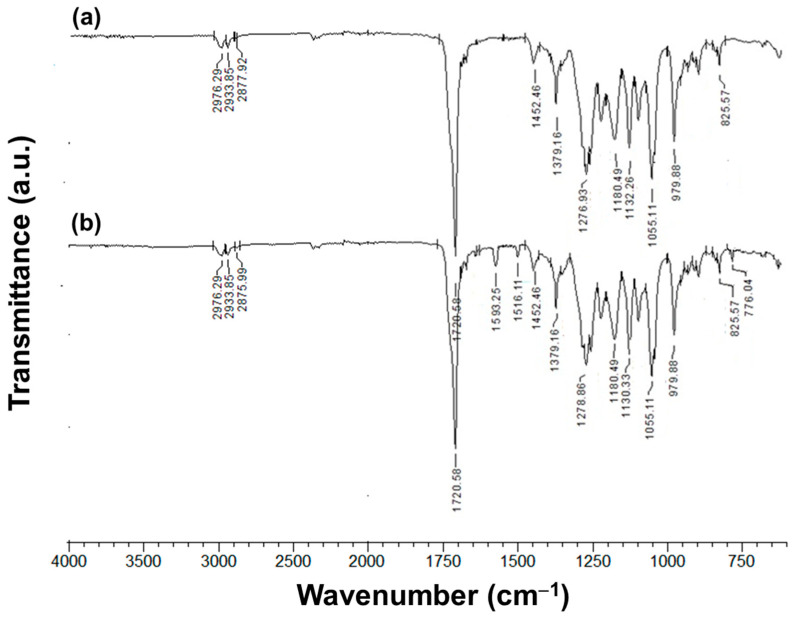

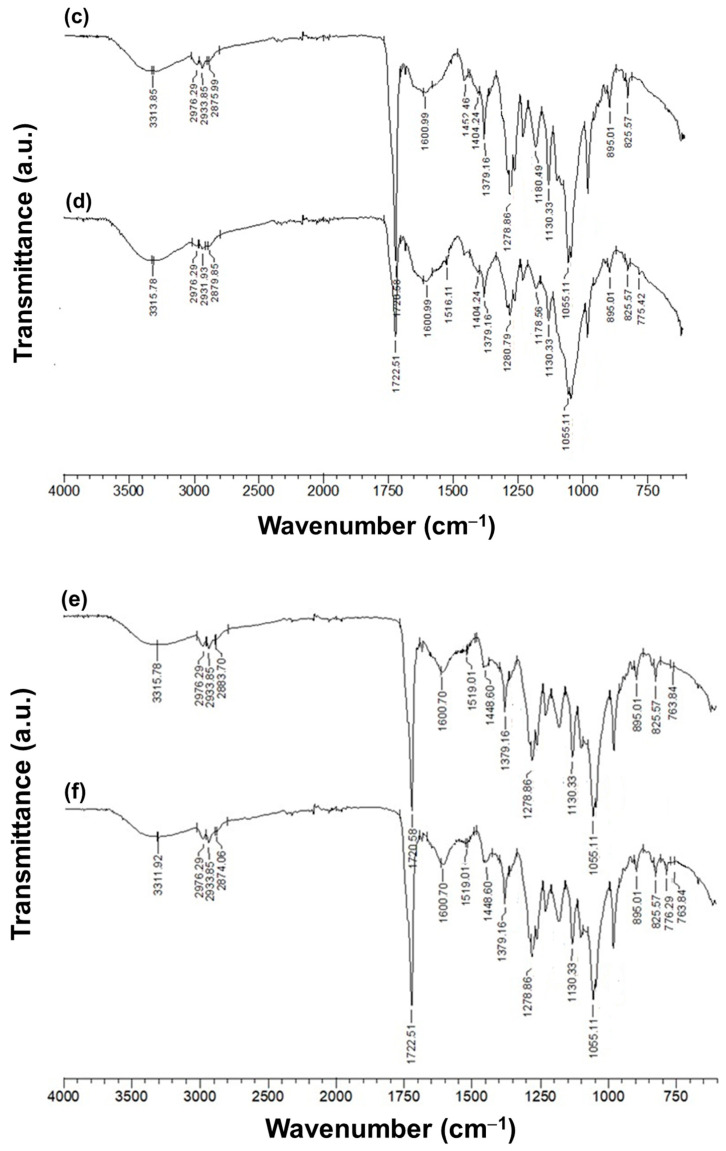
ATR–FTIR spectra of mats: (**a**) PHB; (**b**) MO-*in*-PHB (10 wt.% MO); (**c**) Ch/HA-*coat*-PHB; (**d**) Ch/HA-*coat*-(MO-*in*-PHB) (10 wt.% MO); (**e**) (Ch,HP)/HA-*coat*-PHB (20 wt.% HP); and (**f**) (Ch,HP)/HA-*coat*-(MO-*in*-PHB) (10 wt.% MO, 20 wt.% HP).

ATR–FTIR spectroscopy was used to analyze the chemical structure of the mats containing MO and/or HP extracts. In the spectrum of the MO extract, which is a mixture of large number of compounds, a broad band at 3337 cm^−1^ ascribed to the O-H stretching (OH groups (H-bonded)) corresponding to alcohols and phenols [58] was observed, as well as bands at 2982, 2930, and 2895 cm^−1^ for C-H aliphatic stretching vibrations; bands at 1694 cm^−1^ and at 1593 cm^−1^, which can be attributed to C=C and C=O stretching found in phenolic acids and flavonoids; a band at 1516 cm^−1^, which can be ascribed to C=C stretching vibrations from an aromatic ring; and a band at 1387 cm^−1^ corresponding to -C-H bending of functional groups in an alkane [41,59] (Supplementary Materials, Figure S6a). A band at 1047 cm^−1^ was also recorded in the spectrum of the MO extract, which can be assigned to the C-O bending vibration in ester groups or in secondary cyclic alcohols [41,59]. In addition, the bands at 816 and 775 cm^−1^ are due to the out-of-plane bending of the aromatic ring of polyphenols [41]. The spectrum of the PHB mat (Figure 4a) showed absorption bands at 2976, 2934, and 2878 cm^−1^, characteristic of C-H aliphatic stretching; at 1721 cm^−1^, assigned to C=O stretching; at 1452 cm^−1^, related to the C-H bending of the polymer chain; and at 1055 cm^−1^, assigned to C-O-C stretching. In the ATR–FTIR spectrum of MO-*in*-PHB mats (Figure 4b) in addition to the above-mentioned characteristic bands of PHB, absorption bands at 1593 cm^−1^, 1516 cm^−1^, and 776 cm^−1^, respectively, for C=O stretching, C=C stretching of the aromatic ring and out-of-plane bending of the aromatic ring from the phenolic compounds in the MO extract were recorded. These observations suggest the successful loading of MO in the PHB mats.

The spectrum of the Ch/HA-coated PHB mats (Figure 4c) displayed new bands: a broad band at 3314 cm^−1^ assigned to O-H and N-H stretching from the Ch/HA PEC coating, a broad band between 1700 and 1500 cm^−1^ due to the overlapping of the vibrations of carbonyl and amide groups of Ch and HA, with a maximum at 1601 cm^−1^ related to ν_as_(COO^−^) vibrations from the HA of the PEC coating and a band at 1404 cm^−1^ characteristic of the ν_s_(COO^−^) of HA, with the exception of the above-mentioned characteristic bands of PHB. The spectrum of Ch/HA-*coat*-(MO-*in*-PHB) mats showed characteristic bands assigned to PHB, the MO extract, and the Ch/HA PEC coating (Figure 4d). In addition, the band for ν_as_(COO^−^), which was detected in the spectrum of free HA at 1609 cm^−1^ (Supplementary Materials, Figure S6d), was shifted bathochromically by 8 cm^−1^ at 1601 cm^−1^ in the spectra of Ch/HA PEC (Supplementary Materials, Figure S6e) and PHB or MO-*in*-PHB mats coated with Ch/HA (Figure 4c,d). This indicated that ionic interactions between the carboxylate groups of HA and protonated amino groups of Ch in Ch/HA PEC and in Ch/HA PEC coating on the fiber surface occurred.

The spectrum of the ethanolic extract of HP (Supplementary Materials, Figure S6b), consisting of a variety of secondary metabolites, revealed absorption bands at 3277 cm^−1^ that were ascribed to the O-H stretching; at 2969, 2926, and 2857 cm^−1^, characteristic for the stretching of C-H; at 1716 cm^−1^, attributed to the C=O vibration of the -C-(C=O)-C- group; at 1601 cm^−1^, assigned to the stretching of the carbonyl group as well as C=C stretching; at 1516 cm^−1^, characteristic of C=C aromatic stretching; at 1445 cm^−1^, due to C-H bending; at 1373 cm^−1^, corresponding to C=C aromatic stretching as well as the bending of the methyl group; and at 1202 cm^−1^, specific of the C-O phenolic groups. An absorption band corresponding to the C-O-C stretching was also recorded in the spectrum of the HP extract at 1053 cm^−1^. The detected bands at 818 and 768 cm^−1^ were ascribed to the out-of-plane bending vibration of aromatic rings. In the spectra of (Ch,HP)/HA-*coat*-PHB and (Ch,HP)/HA-*coat*-(MO-*in*-PHB) mats, new characteristic bands at 1519 cm^−1^ and 764 cm^−1^ were recorded (Figure 4e,f). The band at 1519 cm^−1^ was attributed to C=C stretching vibrations of aromatic rings, and the band at 764 cm^−1^ was related to the out-of-plane bending of aromatic rings from the secondary metabolites, which were present in the HP extract. All other characteristic bands of the HP extract overlapped with the vibrations of functional groups from PHB, the Ch/HA PEC coating, or the MO extract. These results provided proof for the presence of HP extract in the coated mats.

### 3.5. XRD Analysis of the Mats

Figure S7 (Supplementary Materials) displays the XRD patterns of PHB, MO-*in*-PHB, PEC-coated PHB and MO-*in*-PHB mats, (Ch,HP)/HA-*coat*-PHB, (Ch,HP)/HA-*coat*-(MO-*in*-PHB) mats, and MO and HP extract powder registered from 5 to 60°. PHB is a semi-crystalline aliphatic polyester possessing well-defined diffraction peaks at around 13°, 16°, 21°, 22°, 25°, and 27° [60]. These diffractions attributed to the crystalline phase of PHB are present in the XRD patterns of non-coated and PEC-coated mats containing MO and/or HP (Figure S7, Supplementary Materials). In the XRD patterns of MO and HP extracts, the diffraction peaks were missing, which indicated that the extracts were amorphous (Figure S7a,d, Supplementary Materials). It has been reported by other authors that Ch/HA PEC had an amorphous structure [61]. In all XRD patterns of the fibrous mats, with the exception of the diffractions due to PHB crystallinity, no additional diffraction peaks were recorded. Most likely, after the incorporation of the extracts into the fibers or into the coating, the low-molecular weight compounds contained in the extracts do not crystallize.

### 3.6. Thermal Behavior of the Mats

TGA was carried out to assess the thermal stability of the fabricated mats. For comparison, the TGA curves of MO and HP extracts are also presented in Figure S8a,b (Supplementary Materials). In the case of the MO extract, weight loss was observed in four stages in the temperature ranges of 50–120 °C (3.6%), 120–250 °C (16.1%), 250–350 °C (16.1%), and 350–450 °C (22.7%). The initial weight loss was due to water evaporation, while the remaining three stages with peaks at 214 °C, 290 °C, and 369 °C corresponded to the degradation of phenolic acids and flavonoids contained in the MO extract [41]. Thermogravimetric data showed that the HP extract degraded in a five-step process. The first step with a weight loss of 5.0% was the result of water evaporation (50–120 °C). The second stage of degradation was in the range of 120–210 °C with a 9.1% weight loss, the third stage was in the range of 210–296 °C with an 11.2% weight loss, the fourth stage was in the range of 296–373 °C (weight loss 10.1%), and the fifth stage was in the range of 373–550 °C (weight loss 23.4%), which arose from the degradation of phenolic acids, flavonoids, hypericin and its derivatives, and phloroglucinols present in the HP extract. As seen from Figure S8g,h (Supplementary Materials), PHB and MO-*in*-PHB mats had a one-step degradation in the 220–350 °C range. The maximum degradation temperature of the PHB mats was 300 °C, while the PHB mats containing MO had a lower value of the maximum degradation temperature at 279 °C. This indicates that the addition of MO to PHB slightly decreases the PHB thermal stability. A drop in the melting temperature has been registered for electrospun systems based on cellulose acetate or poly(L-lactide) loaded with rosmarinic acid, the major component in the MO [62,63]. The authors suggest a decrease in the polymer carrier crystallinity by increasing the content of rosmarinic acid. This could be the reason for the registered lower thermostability of PHB loaded with MO. For the Ch/HA PEC-coated PHB and MO-*in*-PHB mats as well as (Ch,HP)/HA-*coat*-PHB and (Ch,HP)/HA-*coat*-(MO-*in*-PHB) mats, a lower temperature region (50–160 °C) (2.5–4.2% weight loss) was observed due to the evaporation of water, which was attributed to the Ch/HA or (Ch,HP)/HA coating, followed by a one-step degradation process that began at 210 °C and ended at 340 °C. The maximum degradation temperatures were 275 °C, 277 °C, 279 °C, and 276 °C for Ch/HA-*coat*-PHB, (Ch,HP)/HA-*coat*-PHB, Ch/HA-*coat*-(MO-*in*-PHB), and (Ch,HP)/HA-*coat*-(MO-*in*-PHB) mats, respectively. These values are lower than that detected for the PHB mats. The value of the maximum degradation temperature for the Ch/HA complex reported by other authors is around 280 °C [64]. The observed lower thermal stability of these mats in comparison with that of PHB mats was most likely due to the presence of a film coating of Ch/HA as well as Ch/HA containing HP with lower thermal stability on the fiber surface. The obtained data are in conformity with the results of other reports, which show a decrease in the thermal stability of polyethylene terephthalate when it is coated with a nanolayered film of chitosan/alginate [65]. In contrast to the PHB mats, which completely degrade at 800 °C, the residual mass was 3.8 % for the Ch/HA-*coat*-PHB mats and 11.2% for the (Ch,HP)/HA-*coat*-PHB mats at 800 °C. These residues were attributed to the Ch/HA coating and the Ch/HA coating containing HP. The residual mass was 3.7% for the MO-*in*-PHB mats, 7.5% for the Ch/HA-*coat*-(MO-*in*-PHB) mats, and 14.9% for the (Ch,HP)/HA-*coat*-(MO-*in*-PHB) mats at 800 °C. Considering the amount of residue in the degradation of MO (about 41%) and HP (about 41%) extracts at 800 °C and the amount of residue for the mats containing MO and/or HP, it was found that the MO content was 10 wt% and the HP content was 20 wt% with respect to the PHB weight.

### 3.7. In Vitro Release Studies

**Figure 5:**
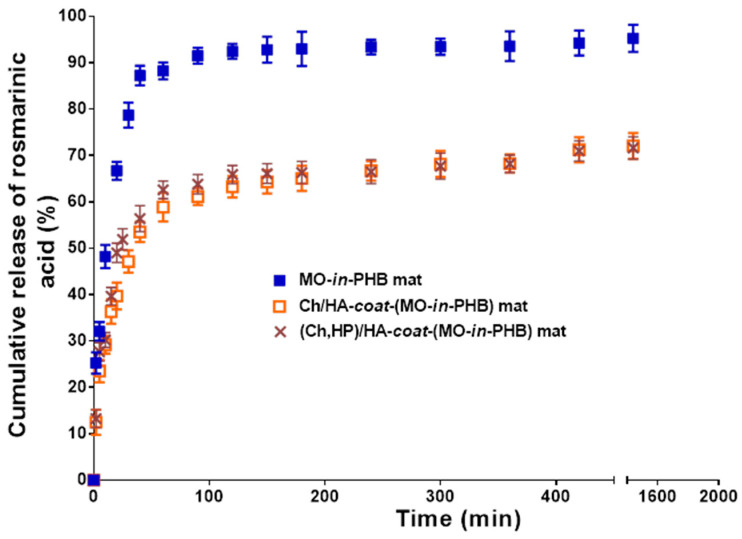
Release profiles of rosmarinic acid from: MO-*in*-PHB mat (10 wt.% MO), Ch/HA-*coat*-(MO-*in*-PHB) mat (10 wt.% MO), and (Ch,HP/HA)-*coat*-(MO-*in*-PHB) mat (10 wt.% MO, 20 wt.% HP) in PBS; pH 7.4; 37 °C.

In terms of the potential biomedical applications of the prepared fibrous materials, it is of interest to investigate the influence of the composition of the materials on the release behavior of the main bioactive compounds found in the MO and HO extracts incorporated in the materials. Therefore, the in vitro release profile of rosmarinic acid, one of the main bioactive compounds present in the MO extract, from the (non-)coated MO-containing mats was investigated by HPLC-DAD-ESI/MS analysis in PBS (Figure 5). As seen from the Figure, each curve has an initial stage of rapid release, which is most likely due to the diffusion of rosmarinic acid, which is placed near the fiber surface. A second stage of gradual release was then observed, and a plateau was reached at 1440 min. We found that the presence of a Ch/HA or (Ch,HP)/HA PEC film on the fiber surface, through which rosmarinic acid incorporated into the bulk of the fibers must diffuse, led to a slower release of rosmarinic acid. The results are consistent with the data provided in our previous reports on PEC-coated fibrous materials loaded with caffeic acid [66] as well as with data from other studies on Ch/pectin PEC-based nanocapsules loaded with indomethacin [67]. Approximately 54% and 56% of the released rosmarinic acid was recorded in the initial 40 min from Ch/HA-*coat*-(MO-*in*-PHB) and (Ch,HP)/HA-*coat*-(MO-*in*-PHB) mats, respectively. For the same time period, the non-coated MO-*in*-PHB mats released 87.2 ± 2.1% rosmarinic acid (Figure 5). The total amount of rosmarinic acid released from MO-*in*-PHB, Ch/HA-*coat*-(MO-in-PHB), and (Ch,HP)/HA-*coat*-(MO-*in*-PHB) mats for 1440 min was 95.3 ± 3.0%, 72.1 ± 2.8%, and 71.6 ± 2.4%, respectively.

**Figure 6:**
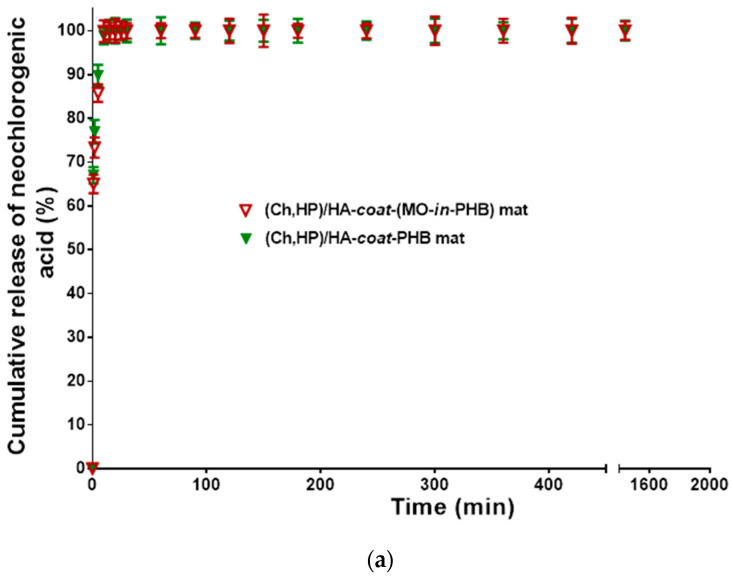

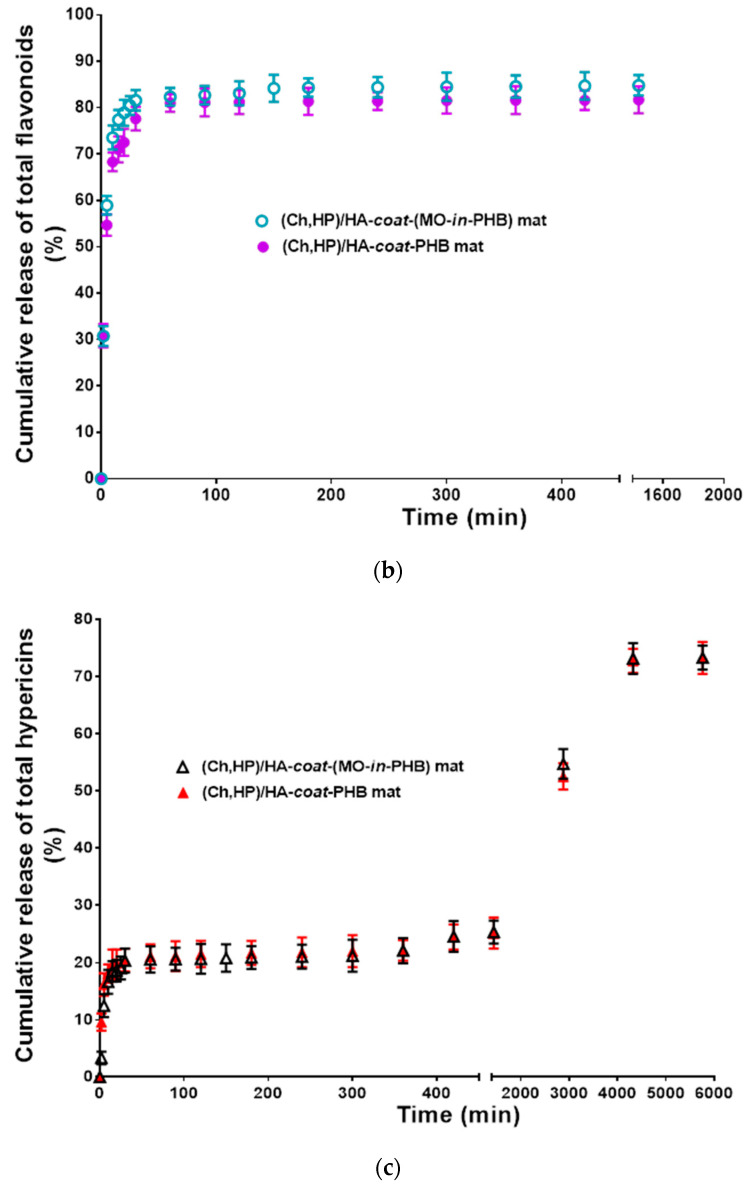
Release profiles of (**a**) neochlorogenic acid, (**b**) total flavonoids, and (**c**) total hypericins from (Ch,HP/HA)-*coat*-(MO-*in*-PHB) mat (10 wt.% MO, 20 wt.% HP) and (Ch,HP/HA)-*coat*-PHB mat (20 wt.% HP) in PBS; pH 7.4; 37 °C.

The in vitro release profiles of neochlorogenic acid, total flavonoids, and total hypericins, the major bioactive compounds found in the HP extract, from the studied mats were also investigated in PBS solution (Figure 6). In vitro release profiles were characterized by an initial stage of burst release followed by a gradual mode of release. Neochlorogenic acid was released the fastest and in the largest amount compared with the total flavonoids and total hypericins from the respective mats. Approximately 100% of the released neochlorogenic acid was recorded in 20 min for the studied mats. The amount of total flavonoids released from the coated PHB and MO-*in*-PHB mats for 20 min was about 73% and 79%, and for 1440 min, it was 82% and 85%, respectively. This release profile of neochlorogenic acid and total flavonoids makes the materials promising as delivery systems for bioactive compounds, which allow us to achieve a rapid biological effect. For the coated fibrous materials containing HP or both extracts, the release of total hypericins was slowest, and in the first stage, the released amount in 30 min was approximately 20%. The maximum released amount of total hypericins from the respective mats reached about 73% in 4320 min. The observed gradual release of total hypericins indicates that the materials are also suitable as systems for prolonged biological effects.

### 3.8. Antioxidant Properties of the Mats

**Figure 7:**
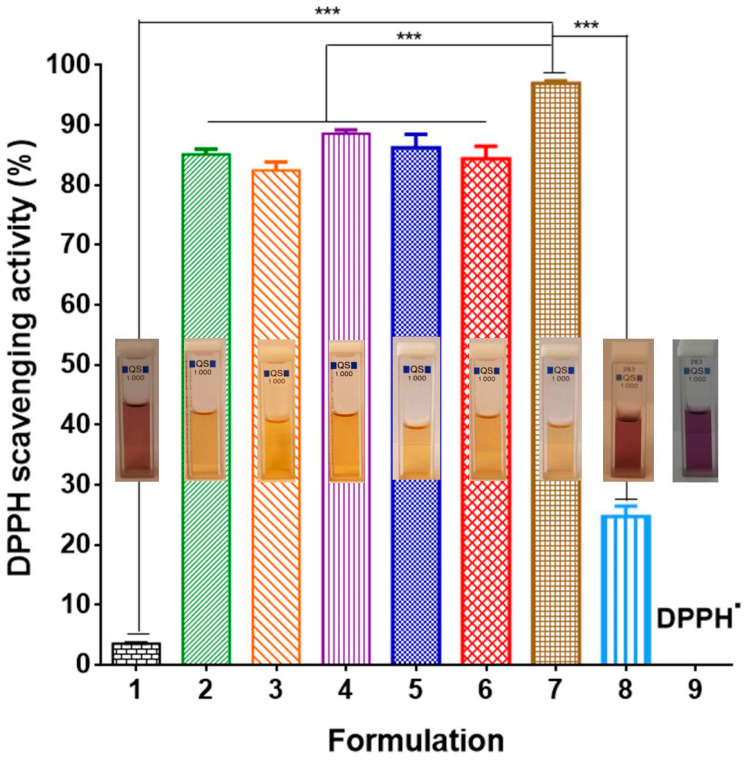
Antioxidant properties of: 1—PHB mat; 2—MO-*in*-PHB mat (10 wt.% MO); 3—extract of MO; 4—Ch/HA-*coat*-(MO-*in*-PHB) mat (10 wt.% MO); 5—(Ch,HP)/HA-*coat*-PHB mat (10 wt.% HP); 6—extract of HP; 7—(Ch,HP)/HA-*coat*-(MO-*in*-PHB) mat (10 wt.% MO, 10 wt.% HP); and 8—Ch/HA-*coat*-PHB mat. *** *p* < 0.001. The solution of DPPH• is also presented (9). Digital images of the solutions are displayed.

It has been demonstrated that MO, HP, HA, and Ch manifest antioxidant activity [26,37,68,69]. It can be assumed that the incorporation of extracts in mats or the deposition of extracts and PEC on the mat surface will provide them with antioxidant potential. Therefore, the antioxidant capacity of MO-loaded PHB mats as well as PEC-coated PHB and MO-*in*-PHB mats (not) containing HP was estimated by the DPPH• scavenging test (Figure 7). In this test, the absorbance of DPPH radical dot’s solution at 517 nm with the addition of the fabricated materials within 30 min of incubation was recorded via a UV-Vis spectrophotometer. In comparison, the antioxidant potential of PHB mats was detected as well. It was found that these mats possessed very low antioxidant activity. Within 30 min, the absorbance of DPPH• dropped to 3.60 ± 0.08% for PHB mats. In contrast, for the same time of contact, a significant decline in the absorbance of DPPH• (by 85.10 ± 0.93%) was recorded for the PHB mats loaded with MO. Contact with a solution of MO extract resulted in a drop in DPPH• solution absorption (by 82.45 ± 1.45%) that was similar to that observed in the case of contact with PHB mats loaded with MO (Figure 7). Ch/HA-coated MO-*in*-PHB mats exhibited a slightly higher effect on the DPPH solutions (absorbance of DPPH• dropped by 88.6%). The DPPH scavenging capacity of Ch/HA- coated PHB mats was 24.75 ± 1.70% (Figure 7). PHB mats coated with Ch/HA containing HP demonstrated higher antioxidant activity (88.25 ± 0.96%). Furthermore, the DPPH• scavenging capacity of PEC-coated mats containing both extracts (97.14 ± 0.67%) was greater than that of (non-)coated MO-loaded PHB mats and PHB mats coated with PEC embedded with HP alone (Figure 7). This fact is most probably a result of the combination of the antioxidant capacity of Ch, HA, and extracts of MO and HP. The results from the DPPH test demonstrated that MO and HP extracts maintained their significant antioxidant activity upon incorporation in the mats or in the coating on the fiber surface.

### 3.9. Antibacterial Activity of the Fibrous Mats

**Figure 8:**
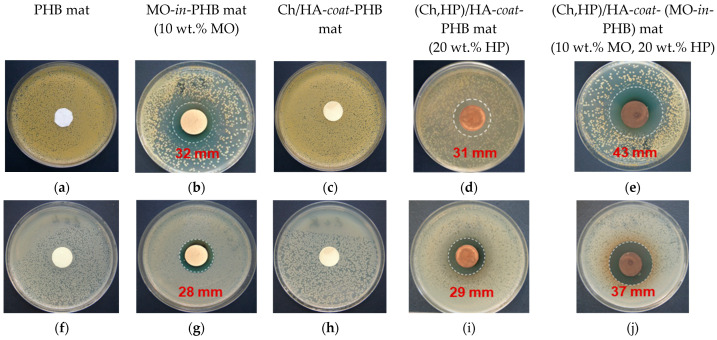
Photographs of the sterile zones towards *S. aureus* (**a**–**e**) and *E. coli* (**f**–**j**), registered around the fibrous mats.

In view of the potential wound-healing application of the obtained non-coated and PEC-coated mats containing MO, HP, or both extracts, the antibacterial activity of these materials against Gram-positive bacteria *S. aureus* and Gram-negative bacteria *E. coli* was evaluated by determining the sterile zones around discs from the materials. In Figure 8, Petri dish images in which discs from the fibrous mats were placed into contact for 24 h with the tested pathogenic bacteria are shown. Non-coated and PEC-coated PHB mats displayed no antibacterial activity and sterile zones around these samples were not registered (Figure 8). In contrast, MO-loaded mats and PEC-coated mats containing HP or both extracts exerted antibacterial activity. A well-distinguished inhibition field was detected around these fibrous discs. The mean diameters of the sterile zones for the MO-*in*-PHB, (Ch,HP)/HA-*coat*-PHB, and (Ch,HP)/HA-*coat*-(MO-*in*-PHB) mats were 32, 31, and 43 mm, respectively, in the *S. aureus* tests. As can be seen, these fibrous materials had a slightly higher impact on the inhibition of *S. aureus* bacteria growth than that of *E. coli* bacteria (Figure 8). This finding is most likely due to the difference in the structure of the cell wall of Gram-positive and Gram-negative bacteria [70,71]. Moreover, the antibacterial activity of the mats containing a combination of MO and HP and the coating of Ch/HA was greater compared with that of MO-loaded mats and (Ch,HP)/HA-coated mats alone (Figure 8). The observed large inhibition zones of the tested MO-*in*-PHB, (Ch,HP)/HA-*coat*-PHB, and (Ch,HP)/HA-*coat*-(MO-*in*-PHB) mats against *S. aureus* and also against *E. coli* give us reason to compare their bactericidal activity with that of bactericidal antibiotic gentamicin, which possess broad spectrum activity against Gram-positive and Gram-negative bacteria [72].

The results from the conducted tests demonstrated that the incorporation of MO into the mats, as well as the loading of HP in the coating on the surface of the mats or the combination of both extracts, imparted high antibacterial activity to the fibrous materials. The mechanism governing the action of MO and HP extracts in bacteria cells is still unknown. It is considered that the antibacterial efficacy of MO extract is mainly due to the disruption of cell wall and membrane integrity, leading to nucleic acid and protein efflux, thus inhibiting the growth of bacteria [73]. Some authors suggest that extract of HP causes cell shrinkage and distortion in bacteria cells and subsequently causes cytoplasmic leakage by attaching to the enzymes and proteins, resulting in the cell death of bacteria [74].

### 3.10. Evaluation of the Effect of the Fibrous Mats on the Viability of HeLa and BALB/3T3 Cells Using an MTT Assay

The in vitro anticancer effects of the MO and HP extracts on HeLa cervical carcinoma cells were evaluated by an MTT test and compared with that of the standard anthracycline cytostatic agent DOX. The IC_50_ values were calculated and are presented in Table 2.

As evident from the presented data, the standard cytostatic drug showed higher cytotoxicity towards the studied cancer cells as compared with MO and HP. DOX is a widely used anticancer chemotherapeutic agent applied in clinical practice for the treatment of various types of cancer. However, the therapeutic application of DOX is restricted by its severe toxic effects to non-tumor cells [75]. The advantage of MO and HP extracts as potential anticancer agents is their selective activity towards cancer cells, as confirmed by the results of previous studies [76,77].

**Figure 9:**
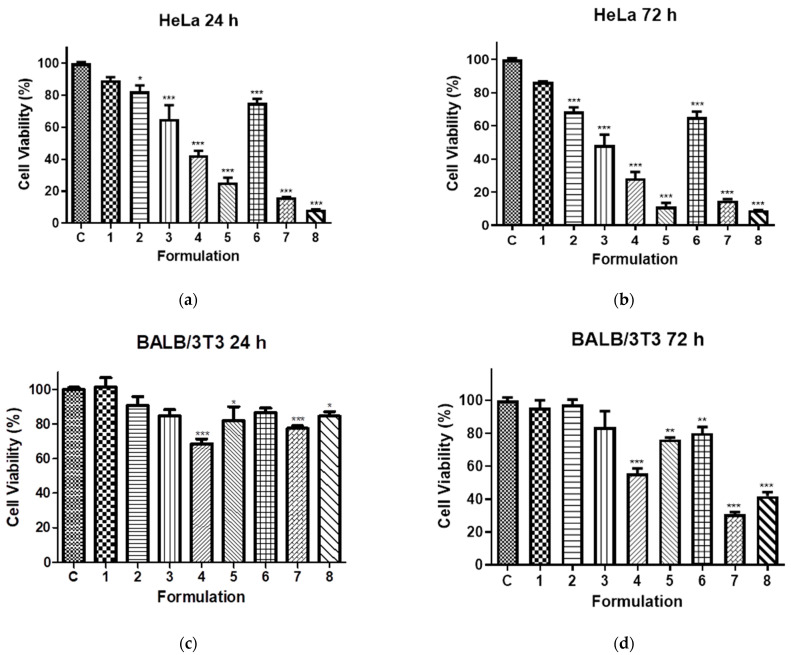
Viability of HeLa or BALB/3T3 cells after 24 (**a**,**c**) and 72 h (**b**,**d**) when in contact with: C—HeLa or fibroblast cells (control); 1—PHB mat; 2—Ch/HA-*coat*-PHB mat; 3—MO-*in*-PHB mat; 4—(Ch,HP)/HA-*coat*-PHB mat; 5—(Ch,HP)/HA-*coat*-(MO-*in*-PHB) mat; 6—solution of MO extract; 7—solution of HP extract; and 8—solution of a mixture of MO and HP extracts (1:2 *w*/*w*). All samples containing MO and HP were investigated at a concentration of MO and HP of 130 μg/mL and 260 μg/mL, respectively. * *p* < 0.05, ** *p* < 0.01, *** *p* < 0.001.

The effect of the prepared fibrous materials containing MO, HP, or both extracts on the viability of the HeLa cancer cell line was also estimated in vitro by the MTT cytotoxicity assay. The MO, HP, and mixture of MO and HP extracts were used as positive controls. As evident from Figure 9a, when the cells were treated with PHB mats for 24 h, an insignificant decrease in the percentage of HeLa cell viability was detected. The Ch/HA-*coat*-PHB mats exerted a slightly statistically significant antiproliferative effect (82.5 ± 9.2% viability) against HeLa cells. In contrast to them, after 24 h of incubation, MO-loaded PHB mats and PEC-coated PHB mats containing HP exerted a considerable inhibitory effect on the viability of HeLa cells; the viability of the cells dropped down to approx. 65% and 42% for the respective mats (Figure 9a). The greatest anticancer effect among the fibrous materials against HeLa cells was registered for materials containing the combination of both extracts and a Ch/HA complex (approx. 25% viability). As seen from the presented data, the most prominent antiproliferative effects of fibrous materials containing MO, HP, or both extracts were established after 72 h of incubation (Figure 9b). It was found that the materials that contained MO and HP extracts and a Ch/HA coating (11.2 ± 5.6% viability) displayed stronger cytotoxicity than MO-*in*-PHB (48.5 ± 14.1% viability) and (Ch,HP)/HA-*coat*-PHB mats (28.3 ± 9.7% viability) alone. Similarly, the solution of a mixture of MO and HP extracts (approx. 9% viability) manifested higher anticancer efficacy than free MO (approx. 65% viability) and free HP (approx. 15% viability). Moreover, the cell viability was reduced to approx. 69% when PHB mats were coated with Ch/HA (Figure 9b).

An assessment of the cytotoxicity of the tested novel materials on the BALB/3T3 non-cancer mouse fibroblast cell line was performed as well. A statistically significant reduction in the viability of BALB/3T3 cells at the 24th hour was detected after treatment with PEC-coated mats containing HP (approx. 69% viability) or both extracts (approx. 82% viability) and after treatment with solutions of HP (approx. 78% viability) or a mixture of MO and HP (approx. 85% viability) (Figure 9c). The (non-)coated PHB and MO-loaded PHB mats exhibited no cytotoxicity against tested non-cancer cells, even after 72 h of incubation (Figure 9d). At the 72nd hour, a decrease in the non-cancer cell viability induced in the presence of (Ch,HP)/HA-*coat*-PHB (approx. 56% viability) and (Ch,HP)/HA-*coat*-(MO-*in*-PHB) (approx. 76% viability) mats was lower than that recorded for cancer cells (Figure 9b,d). The acquired results revealed that the fabricated extract-containing materials exerted a strong inhibitory effect on the growth of HeLa cancer cells while manifesting a lower level of toxicity towards non-cancer BALB/3T3 fibroblasts.

### 3.11. Fluorescence Microscopy Analysis of Cell Death

**Figure 10:**
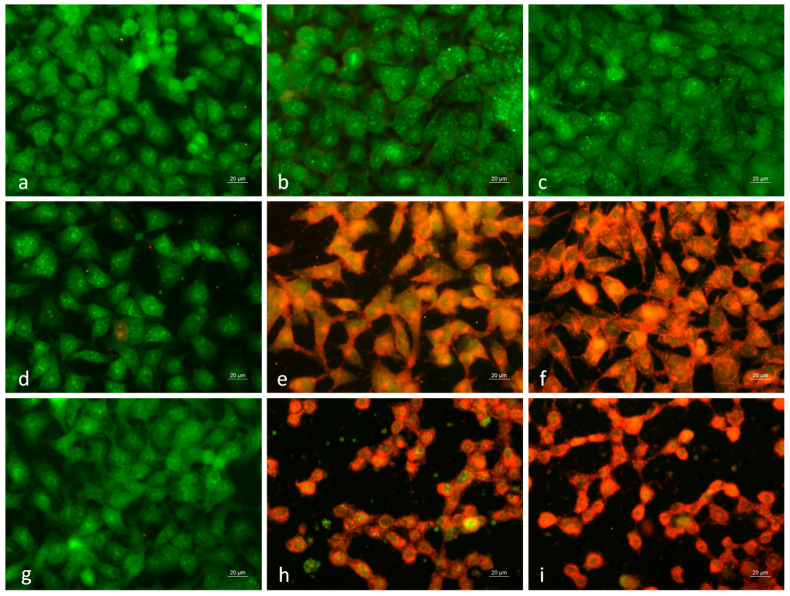
Fluorescence images of HeLa cancer cells stained with AO/EtBr after 24 h of incubation with: (**a**) HeLa cells (control); (**b**) PHB mat; (**c**) Ch/HA-*coat*-PHB mat; (**d**) MO-*in*-PHB mat; (**e**) (Ch,HP)/HA-*coat*-PHB mat; (**f**) (Ch,HP)/HA-*coat*-(MO-*in*-PHB) mat; (**g**) solution of MO extract; (**h**) solution of HP extract; and (**i**) a solution of a mixture of MO and HP extracts (1:2 *w*/*w*). Bar = 20 µm. All samples containing MO and HP were investigated at a concentration of MO and HP of 130 μg/mL and 260 μg/mL, respectively.

To elucidate whether the observed decrease in HeLa cancer cell viability caused by the studied materials is related to the induction of apoptosis, the alterations in cell morphology were investigated by fluorescence microscopy after double staining with AO and EtBr. As shown in Figure 10, there were no alterations in the morphology of control untreated HeLa cells, and HeLa cells cultivated in the presence of PHB mats and a monolayer of uniformly green-stained cells with pale green nuclei containing yellow-green nucleoli was observed (Figure 10a,b). The presence of cells in the phase of mitosis was recorded as well. In the case of the cell culture in the presence of Ch/HA-*coat*-PHB mats (Figure 10c), the number of mitotic cells was slightly reduced and single cells with more intense non-homogeneous green staining of the nucleus were found, indicating an initiation of an apoptotic process. HeLa cells exposed to MO-*in*-PHB mats showed a slightly reduced cell monolayer confluency and the presence of cells with signs of early apoptosis (cell membrane blebbing and chromatin condensation in the nucleus) (Figure 10d). Significant alterations in the cell morphology, indicative of the induction of cell death by apoptosis, were observed when cancer cells were cultivated in the presence of (Ch,HP)/HA-*coat*-PHB and (Ch,HP)/HA-*coat*-(MO-*in*-PHB) mats (Figure 10e,f). In these cases, red staining of the cytoplasm was detected, and it was most intensive in the perinuclear region, in which subcellular membrane structures such as the Golgi apparatus, endoplasmic reticulum, and lysosomes are located.

The nucleus of cancer cells cultivated in the presence of (Ch,HP)/HA-*coat*-PHB mats was green in color but had condensed and aggregated chromatin, which is a morphological feature of early apoptosis (Figure 10e). More severe changes in cells were detected after their treatment with (Ch,HP)/HA-*coat*-(MO-*in*-PHB) mats. Cytoplasmic red staining was more intensive, and most nuclei had strongly red-orange-colored aggregated and condensed chromatin (signs of late apoptosis) (Figure 10f). When the cancer cells were placed in contact with solutions of the extracts, slight alterations in cells were observed after treatment with a solution of MO extract, and severe damage in cells was detected after treatment with solutions of an HP extract and a mixture of MO and HP extracts. In these cases, green-stained cells with early apoptosis changes (Figure 10g) and strongly red-stained cells with signs of late apoptosis (Figure 10h,i) were observed, respectively. The observed red cytoplasmic staining of the cancer cells cultivated in the presence of (Ch,HP)/HA-*coat*-PHB and (Ch,HP)/HA-*coat*-(MO-*in*-PHB) mats and solutions of free HP and a mixture of MO and HP could be explained by the presence of hypericin in the studied formulation composition. Hypericin is a plant pigment present in HP extracts, and it is recognized as one of the main bioactive compounds responsible for their anticancer effects. It exhibits a bright red fluorescence in the range of 600–650 nm [78]. Due to its highly lipophilic nature, hypericin penetrates passively by diffusion through the cell membrane and accumulates in the membranes of different organelles, including lysosomes, mitochondria, the endoplasmic reticulum, and the Golgi apparatus [79]. Moreover, it has been found that the accumulation of hypericin in neoplastic tissue is significantly higher than in normal tissue. In addition, hypericin strongly associates with various proteins such as albumin, low-density lipoprotein, amyloid fibrils, and macromolecules like poly(vinyl pyrrolidone), thus forming red fluorescent conjugates [80,81].

The involvement of apoptosis in the mechanism of action of the studied fibrous mats towards HeLa cells was further investigated by analyzing morphological changes in the nucleus after DAPI staining (Supplementary Materials, Figure S9). The acquired results clearly confirm the data obtained from the AO/EtBr fluorescence analysis (for the analysis of HeLa cell death by the DAPI staining method, see the Supplementary Materials).

The morphology of normal BALB/3T3 mouse fibroblasts cultivated in the presence of the fibrous polymeric materials was examined by fluorescence assays as well. The results obtained from the AO/EtBr staining are presented in Figure S10 (Supplementary Materials). It should be noted that the detected changes in the morphology of the non-cancer cells cultured in the presence of mats containing HP or a combination of both extracts were less prominent than that observed in the HeLa cancer cells. These changes are characteristic of the early phase of apoptosis. The morphological alterations at the cells level observed after treatment of the non-cancer cells with (Ch,HP)/HA-*coat*-(MO-*in*-PHB) mats were weaker compared with those detected for (Ch,HP)/HA-*coat*-PHB mats (Supplementary Materials, Figure S10e,f). PHB mats loaded with MO did not affect the non-cancer cell morphology (Supplementary Materials, Figure S10d).

Fluorescence microscopy images of untreated and treated mouse BALB/3T3 fibroblasts stained with DAPI staining are shown in Figure S11 (Supplementary Materials). The presented data are in accordance with the results of the AO/EtBr study (for the analysis of the nuclear morphological changes of BALB/3T3 cells by the DAPI staining method, see the Supplementary Materials).

The results acquired from fluorescence microscopy analyses were compliant with the data from the conducted MTT test. They showed that the level of cytotoxicity of extract-containing fibrous mats towards HeLa cancer cells was greater than that towards non-cancer mouse BALB/3T3 fibroblasts. The PHB mats loaded with MO had no statistically significant cytotoxic effect on the non-cancer cells. The considerable changes in the morphology found in HeLa cancer cells that were exposed to the prepared fibrous materials demonstrated that the cell death induced by these materials occurred via apoptosis. The recorded high antiproliferative efficacy of (Ch,HP)/HA-*coat*-(MO-*in*-PHB) mats towards cancer cells might have resulted from the combination of the activity of Ch/HA coating and MO and HP extracts.

## 4. Conclusions

In the present study, the fabrication of targeted-design fibrous materials containing MO, HP, or both extracts was achieved effectively by one-pot electrospinning or by combining electrospinning, dip coating, and PEC formation. The morphology, wettability, and structural and thermal characteristics of the developed non-woven mats were thoroughly examined. It was found that the prepared materials with targeted designs provide a possibility for the fast or prolonged release of the main bioactive compounds found in extracts, which was a prerequisite for a fast or continuous biological effect. MO- and/or HP-containing fibrous materials displayed a good capability to induce the growth inhibition of the pathogenic bacteria *S. aureus* and *E. coli*. The measured sterile zones for the MO-*in*-PHB, (Ch,HP)/SH-*on*-PHB and (Ch,HP)/SH-*on*-(MO-*in*-PHB) mats were around 32, 31, and 43 mm, respectively, in the *S. aureus* tests and around 28, 29, and 37 mm in the *E. coli* tests, respectively. The efficacy is most pronounced in the case of materials that contain both MO and HP. The highest DPPH• scavenging activity (97.14 ± 0.67%) was detected for PEC-coated MO-*in*-PHB mats containing HP. The prepared mats manifested low toxicity towards non-cancer mouse BALB/3T3 cells while exhibiting good antiproliferative efficacy against HeLa cancer cells. The detected high anticancer effect of the materials was mainly attributed to the induction of cell apoptosis, as evidenced by the conducted fluorescence microscopy analyses. The presence of two extracts and a Ch/HA coating in the fibrous materials resulted in the highest drop in cancer cell viability. This was most probably due to the combination of the anticancer properties of MO, HP, Ch, and HA. Therefore, the obtained innovative fibrous materials are prospective candidates for dressings in the therapy of wound infections as well as for use in the local therapy of cervical cancer.

## Figures and Tables

**Table 1:** Composition of fabricated fibrous mats.

Fabricated Fibrous Mats	Components			
	PHB	MO	Ch/HA	Ch/HA Loaded with HP
PHB	×			
MO-*in*-PHB	×	×		
Ch/HA-*coat*-PHB	×		×	
Ch/HA-*coat*-(MO-*in*-PHB)	×	×	×	
(Ch,HP)/HA-*coat*-PHB	×			×
(Ch,HP)/HA-*coat*-(MO-*in*-PHB)	×	×		×

Abbreviations: PHB mat is poly(3-hydroxybutyrate) mat prepared by electrospinning; MO-*in*-PHB mat is a mat of PHB loaded with MO encapsulated in the core, prepared by electrospinning; Ch/HA-*coat*-PHB mat is a Ch/HA-coated PHB mat prepared by electrospinning, dip coating, and PEC formation; Ch/HA-*coat*-(MO-*in*-PHB) mat is a Ch/HA-coated MO-*in*-PHB mat prepared by electrospinning, dip coating, and PEC formation; (Ch,HP)/HA-*coat*-PHB mat is a PHB mat coated with Ch/HA loaded with HP prepared by electrospinning, dip coating, and PEC formation; (Ch,HP)/HA-*coat*-(MO-*in*-PHB) mat is an MO-*in*-PHB mat coated with Ch/HA loaded with HP prepared by electrospinning, dip coating, and PEC formation.

**Table 2:** IC_50_ values (µg/mL) of the MO and HP extracts and the standard anticancer drug DOX, as determined by an MTT test on the HeLa cell line after 24 and 72 h of treatment.

Tested Compounds	24 h	72 h
MO	163.2	141.6
HP	182.4	163.2
DOX	2.3	0.1

## Data Availability

The original contributions presented in the study are included in the manuscript, further inquiries can be directed to the corresponding author.

## Supplementary Materials

The following supporting information can be downloaded at: https://www.mdpi.com/article/10.3390/polym16152105/s1, Table S1: The gradient of chromatographic separation; Table S2: Qualitative and quantitative determination of secondary metabolites in HP extract; Figure S1: ^1^H NMR (CD_3_OD, 400 MHz) of I3,II8-biapigenin (**12**); Figure S2: ^1^H NMR (CD_3_OD, 400 MHz) of hyperforin (**17**); Figure S3: COSY (**A**), HSQC (**B**), and HMBC (**C**) spectra of hyperforin (**17**); Figure S4: HPLC-DAD chromatogram of HP extract at 320, 350, 590, and 290 nm; Figure S5: Structures of the main compounds identified in HP extract; Figure S6: ATR–FTIR spectra of: (**a**) extract of MO; (**b**) extract of HP; (**c**) Ch; (**d**) HA and (**e**) PEC Ch/HA; Figure S7: XRD patterns of: (**a**) MO extract; (**b**) PHB mat; (**c**) MO-*in*-PHB mat (10 wt.% MO); (**d**) HP extract; (**e**) Ch/HA-*coat*-PHB mat; (**f**) Ch/HA-*coat*-(MO-*in*-PHB) mat (10 wt.% MO); (**g**) (Ch,HP)/HA-*coat*-PHB mat (20 wt.% HP); and (**h**) (Ch,HP)/HA-*coat*-(MO-*in*-PHB) mat (10 wt.% MO, 20 wt.% HP); Figure S8: TGA thermograms of: (**a**) extract of MO; (**b**) extract of HP; (**c**) (Ch,HP)/HA-*coat*-(MO-*in*-PHB) mat (10 wt.% MO, 20 wt.% HP); (**d**) Ch/HA-*coat*-(MO-*in*-PHB) mat (10 wt.% MO); (**e**) (Ch,HP)/HA-*coat*-PHB mat (20 wt.% HP); (**f**) Ch/HA-*coat*-PHB mat; (**g**) MO-*in*-PHB mat (10 wt.% MO); and (**h**) PHB mat; Figure S9: Fluorescence images of HeLa cancer cells stained with DAPI after 24 h of incubation with: (**a**) HeLa cells (control); (**b**) PHB mat; (**c**) Ch/HA-coat-PHB mat; (**d**) MO-in-PHB mat; (**e**) (Ch,HP)/HA-coat-PHB mat; (**f**) (Ch,HP)/HA-coat-(MO-in-PHB) mat; (**g**) solution of MO extract; (**h**) solution of HP extract and (**i**) solution of a mixture of MO and HP extracts (1:2 *w*/*w*). All samples containing MO and HP were investigated at a concentration of MO and HP-130 μg/mL and 260 μg/mL, respectively; Figure S10. Fluorescence images of BALB/3T3 cells stained with AO/EtBr after 24 h of incubation with: (**a**) BALB/3T3 cells (control); (**b**) PHB mat; (**c**) Ch/HA-coat-PHB mat; (**d**) MO-in-PHB mat; (**e**) (Ch,HP)/HA-coat-PHB mat; (**f**) (Ch,HP)/HA-coat-(MO-in-PHB) mat; (**g**) solution of MO extract; (**h**) solution of HP extract and (**i**) solution of a mixture of MO and HP extracts (1:2 *w*/*w*). All samples containing MO and HP were investigated at a concentration of MO and HP-130 μg/mL and 260 μg/mL, respectively; Figure S11. Fluorescence images of BALB/3T3 cells stained with DAPI after 24 h of incubation with: (**a**) BALB/3T3 cells (control); (**b**) PHB mat; (**c**) Ch/HA-coat-PHB mat; (**d**) MO-in-PHB mat; (**e**) (Ch,HP)/HA-coat-PHB mat; (**f**) (Ch,HP)/HA-coat-(MO-in-PHB) mat; (**g**) solution of MO extract; (**h**) solution of HP extract and (**i**) solution of a mixture of MO and HP extracts (1:2 *w*/*w*). All samples containing MO and HP were investigated at a concentration of MO and HP-130 μg/mL and 260 μg/mL, respectively. References [82,83,84] are cited in the supplementary materials.
