# Supplementary material for: Chitosan/Hyaluronate Complex-Coated Electrospun Poly(3-hydroxybutyrate) Materials Containing Extracts from Melissa officinalis and/or Hypericum perforatum with Various Biological Activities: Antioxidant, Antibacterial and In Vitro Anticancer Effects
Source: Polymers (Basel). 2024 Jul 24;16(15):2105. doi: 10.3390/polym16152105 (PMC11313976; doi:10.3390/polym16152105)
Supplement: Supplementary file 1 [file polymers-16-02105-s001.zip › Supplementary Materials v7 2407 (1).pdf]

polymers

Supplementary Materials

Chitosan/Hyaluronate Complex-Coated Electrospun Poly(3-hydroxybutyrate) Materials Containing Extracts from *Melissa officinalis* and/or *Hypericum perforatum* with Various Biological Activities: Antioxidant, Antibacterial and In Vitro Anticancer Effects

Ina Anastasova [1], Milena Ignatova [1,*], Nevena Manolova [1], Iliya Rashkov [1], Nadya Markova [2], Reneta Toshkova [3], Ani Georgieva [3], Mariana Kamenova-Nacheva [4,5], Antoaneta Trendafilova [5], Viktoria Ivanova [5] and Tsvetelina Doncheva [5]

1 Laboratory of Bioactive Polymers, Institute of Polymers, Bulgarian Academy of Sciences, Acad. G. Bonchev St, Bl. 103A, BG-1113 Sofia, Bulgaria; i_anastasova@polymer.bas.bg (I.A.); ignatova@polymer.bas.bg (M.I.); manolova@polymer.bas.bg (N.M.); rashkov@polymer.bas.bg (I.R.)
2 Institute of Microbiology, Bulgarian Academy of Sciences, Acad. G. Bonchev St, Bl. 26, BG-1113 Sofia, Bulgaria; markn@bas.bg
3 Institute of Experimental Morphology, Pathology and Anthropology with Museum, Bulgarian Academy of Sciences, Acad. G. Bonchev St, bl. 25, BG-1113 Sofia, Bulgaria; rtoshkova@bas.bg (R.T.); ageorgieva@bas.bg (A.G.)
4 Laboratory for Extraction of Natural Products and Synthesis of Bioactive Compounds, Research and Development and Innovation Consortium, Sofia Tech Park JSC, 111 Tsarigradsko Shose blvd.,BG-1784 Sofia, Bulgaria; mariana.nacheva@orgchm.bas.bg
5 Institute of Organic Chemistry with Centre of Phytochemistry, Bulgarian Academy of Sciences, Acad. G. Bonchev Str., bl. 9, BG-1113 Sofia, Bulgaria; antoaneta.trendafilova@orgchm.bas.bg (A.T.); viktoria.genova@orgchm.bas.bg (V.I.); tsvetelina.doncheva@orgchm.bas.bg (T.D.)
* Correspondence: ignatova@polymer.bas.bg; Tel.: +359-(0)2-9792239; Fax: +359-(0)2-8700309

Table S1. The gradient of chromatographic separation.

Time (min)	Solvent A (%)	Solvent B (%)	Solvent C (%)
0	90	10	0
8	90	10	0
10	85	15	0
25	70	20	10
35	10	75	15
50	5	80	15
51	0	90	10
55	0	90	10
58	90	10	0
63	90	10	0

Quantification of rosmarinic acid in MO extract

The quantification of rosmarinic acid in MO was performed at the conditions described in Section 2.3.1. The wavelength selected for the quantification was 330 nm.

Identification was accomplished by comparing the retention times (Rt) and UV spectra of the corresponding peak in the sample to those of the standard. The amount of rosmarinic acid was calculated utilizing a calibration curve (1–50 mg/L, r 2 = 0.9999).

Isolation of I3,II8-biapigenin and hyperforin

The aerial parts of the HP species (5 g) were extracted with methanol (100 mL) in an ultrasonic bath for 30 min. After filtration, the extract was concentrated until reduced pressure to give 460 mg of methanol extract. The latter was subjected on CC (Silica gel, 30 g) using $CHCl_3$ as an eluent. The separation was monitored by TLC (Silica gel, Hexane/Ethyl acetate, 9:1) and 8 fractions were collected. Prep. TLC (Silica gel, Hexane/Ethyl acetate, 9:1) of fraction 3 (44 mg) afforded hyperforin (24.1 mg). CC (Silica gel, 15 g) of fraction 6 (200 mg) using $CHCl_3$/methanol mixture with increasing polarity (from 20:1 to 0:1) afforded 5 subfractions (6/1-6/5). Prep. TLC (RP-18, H_2O/methanol, 1:1) of fraction 6/4 (4.1 mg) led to isolation of I3,II8-biapigenin (1.4 mg).

Phytochemical characterization of HP Extract

The characterization of the main compounds in HP dry extract was performed by HPLC-DAD-ESI/MS in negative ionization mode. A total of 18 compounds (Table S2, Figures S4,S5) were identified by comparison of the retention times, UV/Vis and MS data of the compounds with standards or previously published data.

Compounds **1** and **2** were identified as neochlorogenic and chlorogenic acid by their deprotonated molecular ion $[M – H]^-$ at *m/z* 353 and UV absorption maximum at 325 nm and their structure was confirmed by a direct comparison with authentic standards. Compounds **3** and **4** displayed a deprotonated molecular ion $[M – H]^-$ at *m/z* 331 and UV absorption maximum at 311 nm and were tentatively identified as p-coumaroylquinic acid isomers [82]. Six quercetin O-glycosides: miquelianin (**5**, *m*/z 477), rutin (**6**, *m*/z 609), hyperoside (**7**, *m*/z 463), isoquercitrin (**8**, *m*/z 463), quercitrin (**9**, *m*/z 447) and quercetin 3-*O*-(O-acetyl)-hexoside (**10**, *m*/z 505), as well as the aglycon quercetin (**11**, *m*/z 301) were also detected in HP extract. In addition, the dimeric flavone of apigenin, I3,II8-biapigenin (**12**) was identified by the characterisc UV spectrum (λ_{max} 331 nm) and $[M – H]^-$ at *m/z* 537 and its structure was further confirmed by NMR (Supplementary Materials, Figure S1) after its isolation from HP extract. The characteristic of the genus *Hypericum* naphthodianthrone [protopseudohypericin (**13,** *m/z* 521), pseudohypericin (**14**, *m/z* 519), protohypericin (**15**, *m.z* 505) and hypericin (**16**, *m/z* 503)] and phloroglucinol [hyperforin (**17**, *m/z* 537) and adhyperforin (**18**, *m/z* 549)] derivatives were also identified in HP extract by the corresponding deprotonated molecular ions $[M – H]^-$ and the characteristic UV absorption maxima at 590 and 290 nm, respectively [82-84]. The structure of hyperforin (**17**) was also confirmed by NMR (Supplementary Materials, Figures S2, S3). The presence of hypericin was confirmed by comparison with the commercial standard.

Table S2. Qualitative and quantitative determination of secondary metabolites in HP extract.

Peak	Rt [min]	UV (λ_{max}, nm)	$[M-H]^-$ *m/z*	Compound	Identification	Content [mg/g DE]
				**Phenolic acids**		
1	2.87	325	353	Neochlorogenic acid	St	11.18±0.60
2	3.73	325	353	Chlorogenic acid	St	0.73±0.03
3	3.96	311	337	p-Coumaroylquinic acid isomer 1	[82]	nd

4	4.40	311	337	p-Coumaroylquinic acid isomer 2	[82]	nd
				**Flavonoids**		
5	13.04	352, 257	477	Miquelianin (Quercetin 3-O-glucuronide)	St	3.38±0.18
6	18.11	355, 257	609	Rutin (Quercetin 3-O-rutinoside)	St	45.74±0.8
7	19.11	355, 257	463	Hyperoside (Quercetin 3-O-galactoside)	St	14.17±0.38
8	19.99	350, 254	463	Isoquercitrin (Quercetin 3-O-glucoside)	St	5.55±0.15
9	25.5	351, 254	447	Quercitrin (Quercetin 3-O-rhamnoside)	St	2.35±0.07
10	31.17	355, 257	505	Quercetin 3-O-(O-acetyl-hexoside)	[82]	1.86±0.06
11	31.70	370, 255	301	Quercetin	St	3.22±0.12
12	32.94	331, 268	537	I3,II8-Biapigenin	St, NMR	10.52±0.29
				**Naphthodianthrones**		
13	36.82	544, 374	521	Protopseudohypericin	[83]	0.22±0.02
14	37.51	591, 542, 330, 286	519	Pseudohypericin	[82]	1.61±0.06
15	38.92	537, 535	505	Protohypericin	[82]	0.06±0.02
16	40.00	590, 550, 326, 282	503	Hypericin	St	1.01±0.04
				**Phloroglucinol**		
17	41.07	291	537	Hyperforin	St, NMR	33.6±0.59
18	41.76	291	549	Adhyperforin	[82]	3.60±0.14

St – Standard; NMR – Nuclear Magnetic Resonance; nd – not determined.

The content of the main compounds in HP extract was determined by HPLC-DAD method developed by Zeloi et al. (2017) [51]. Among individual compounds, rutin (45.74 ± 0.80 mg/g), hyperoside (14.17 ± 0.38 mg/g), neochlorogenic acid (11.18 ± 0.60 mg/g), and I3,II8-aiapigenin (10.52 ± 0.29 mg/g) were found in amounts higher than 10 mg/g DE (Supplementary Materials, Table S2). The determined content of total hypericins (0.30%), total flavonoids (8.68%) and hyperforin (3.34%) corresponds to the requirements listed in the European Pharmacopeia for a standardized St. John's wort dry extract, i.e. 0.1 – 0.3% of total hypericins, minimum 6.0% of total flavonoids and maximum 6.0% of hyperforin [54].

Analysis of HeLa cell death by DAPI staining method

The involvement of apoptosis in the mechanism of action of the studied fibrous mats towards HeLa cells was further investigated by analyzing morphological changes in the nucleus after DAPI staining (Supplementary Materials, Figure S9). The acquired results clearly confirm the data obtained from AO/EtBr fluorescence analysis. The nuclear morphology of the HeLa cells that were in contact with PHB mats was similar to that of the untreated control cells (Supplementary Materials, Figure S9 a, b). Single nuclei with

early apoptotic changes expressed in nuclear shrinkage and formation of dot-shaped nuclear fragments were found after treatment with Ch/HA-*coat*-PHB mats (Supplementary Materials, Figure S9 c). Cells exposed to MO-*in*-PHB mats and a solution of MO extract also showed some nuclei with specific apoptotic changes such as condensation and margination of chromatin (Supplementary Materials, Figure S9 d, g). The most pronounced changes in the nuclear morphology, characteristic of apoptosis, were observed when the cells were treated with (Ch,HP)/HA-*coat*-PHB and (Ch,HP)/HA-*coat*-(MO-*in*-PHB) mats (Supplementary Materials, Figure S9 e, f), as well as with the solutions of HP extract and a mixture of MO and HP extracts (Supplementary Materials, Figure S9 h, i). The nuclei were reduced in number, with an uneven contour, nonhomogeneously stained, with condensed and aggregated chromatin and many apoptotic bodies (Supplementary Materials, Figure S9 e, f, h, i). However, nuclei after treatment with (Ch,HP)/HA-*coat*-(MO-*in*-PHB) mats were most affected - a greater number of nuclei were in stages of disintegration and formation of multiple apoptotic bodies (Supplementary Materials, Figure S9 f).

Analysis of the morphological changes in the nucleus of BALB/3T3 cells by DAPI staining method

Fluorescence microscopy images of untreated and treated mouse BALB/3T3 fibroblasts stained with DAPI are shown in Figure S11 (Suplementary Materials). The presented data are in accordance with the results of the AO/EtBr study. Typical early apoptotic nuclear changes, were seen in the cells treated with (Ch,HP)/HA-*coat*-PHB and (Ch,HP)/HA-*coat*-(MO-*in*-PHB) mats (Supplementary Materials, Figure S11 e, f). The nuclei of cells that had been in contact with MO-containing PHB mats possessed similar morphology as the nuclei of control cells (Supplementary Materials, Figure S11d). In this case, less nuclei in phases of mitosis were detected.

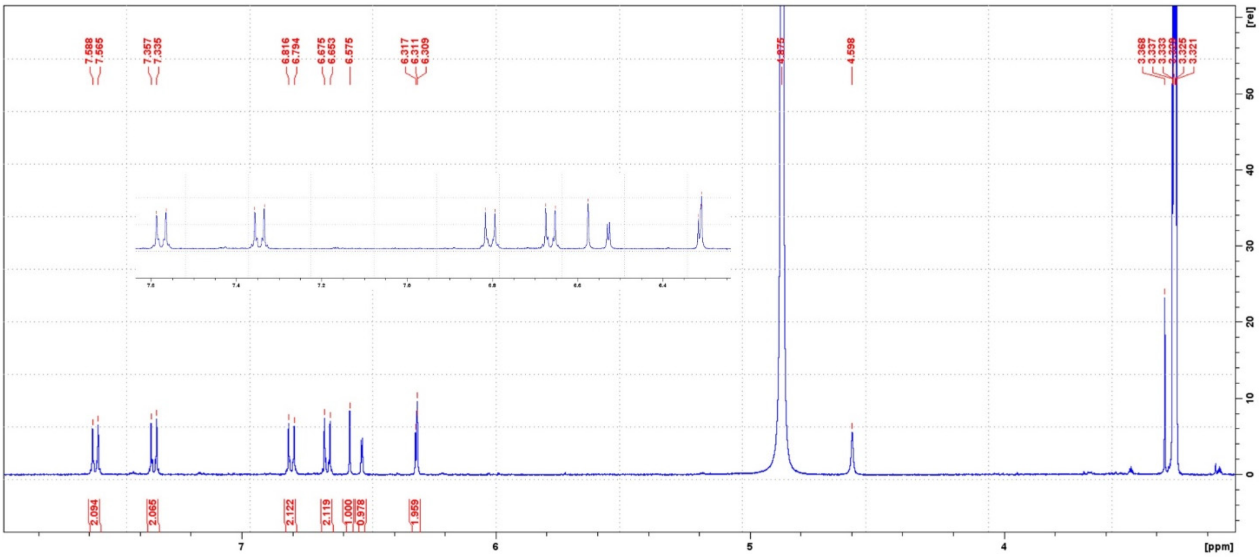

Figure S1. ^{1}H NMR (CD_3OD, 400 MHz) of I3,II8-biapigenin (**12**).

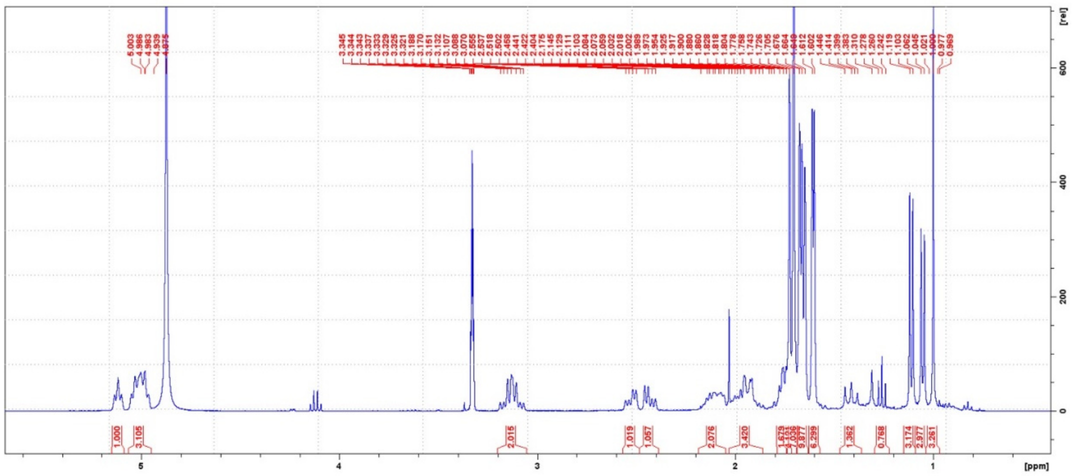

Figure S2. ^{1}H NMR (CD_3OD, 400 MHz) of hyperforin (**17**).

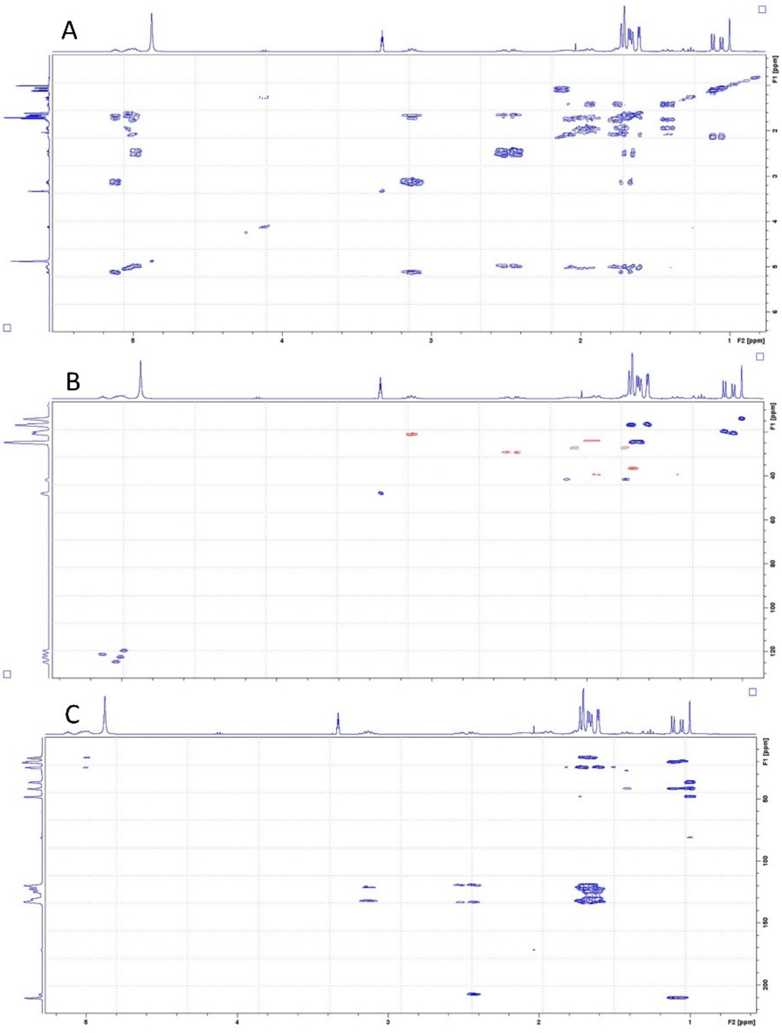

Figure S3. COSY (**A**), HSQC (**B**) and HMBC (**C**) spectra of hyperforin (**17**).

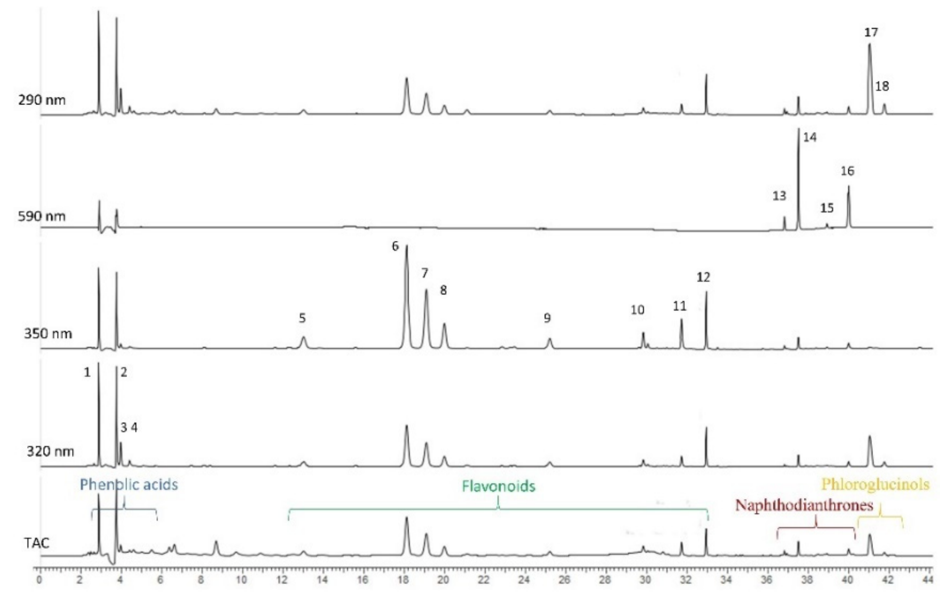

Figure S4. HPLC-DAD chromatogram of HP extract at 320, 350, 590 and 290 nm.

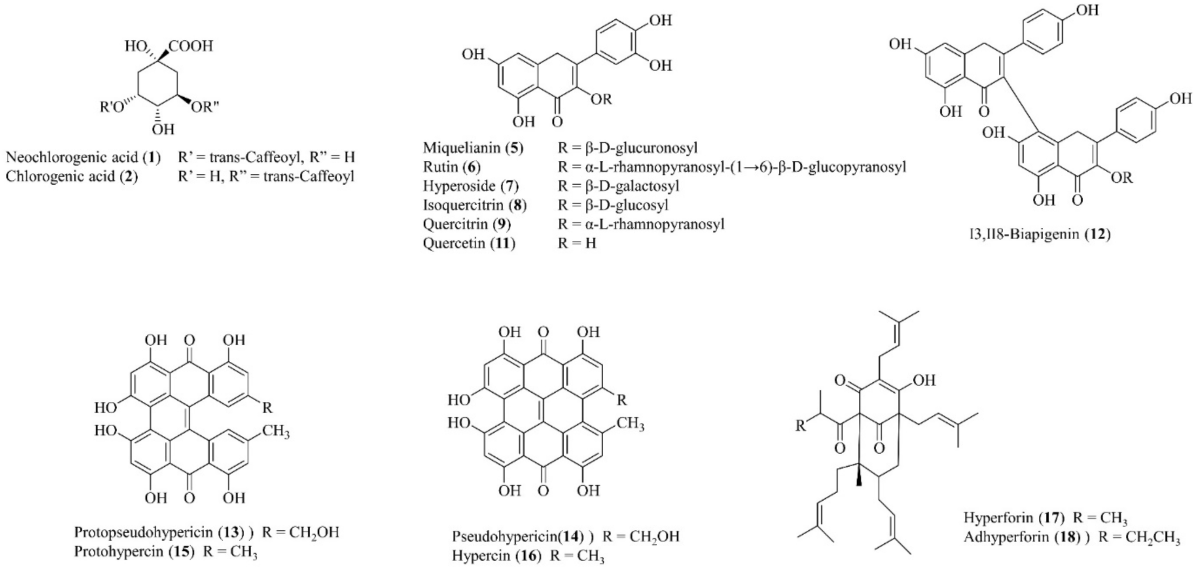

Figure S5. Structures of the main compounds identified in HP extract.

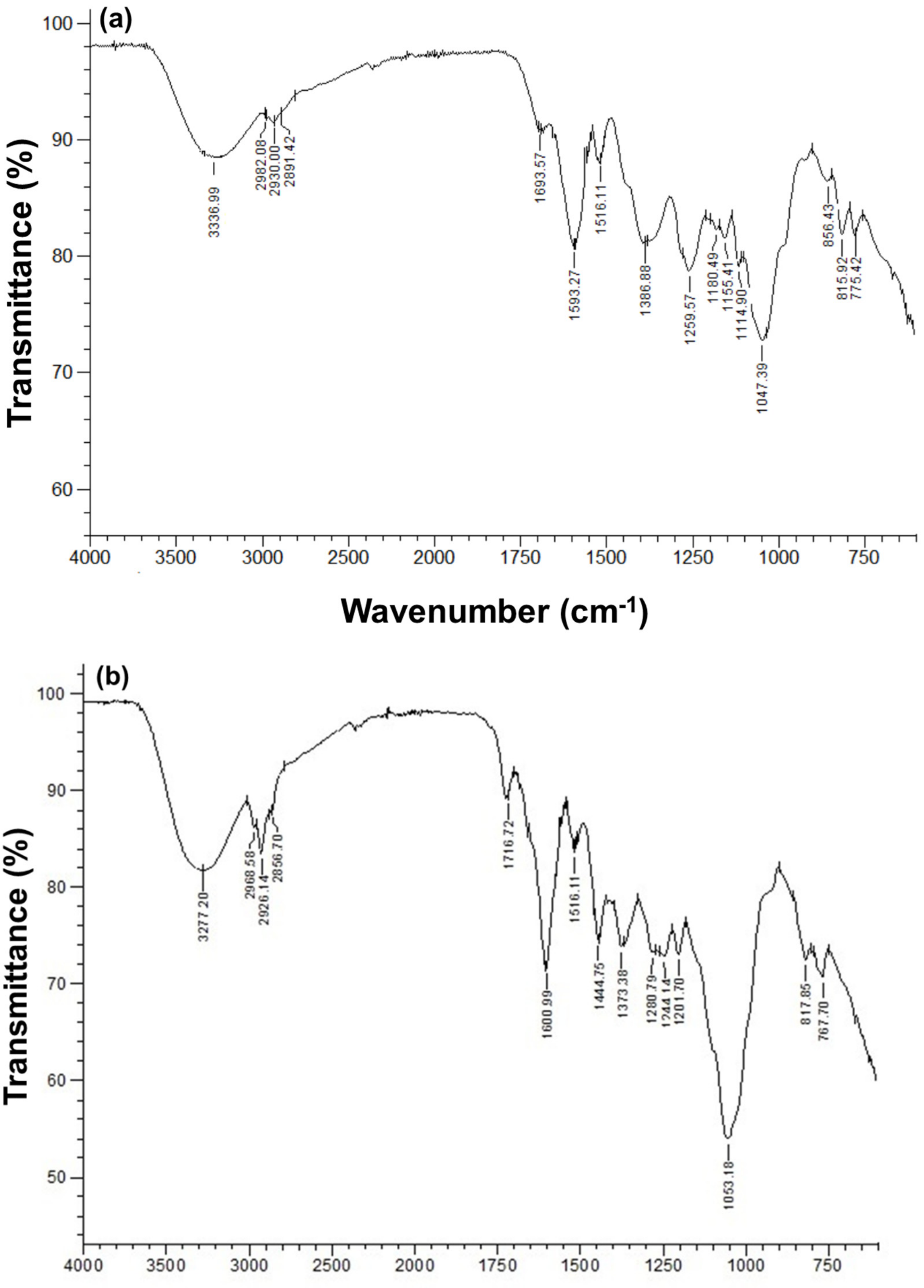

(a)
Transmittance (%)
100
90
80
70
60
3336.99
2982.08
2930.00
2891.42
1693.57
1593.27
1516.11
1386.88
1259.57
1180.49
1155.41
1114.90
1047.39
856.43
815.92
775.42
4000
3500
3000
2500
2000
1750
1500
1250
1000
750
Wavenumber (cm-1)
(b)
Transmittance (%)
100
90
80
70
60
50
3277.20
2968.58
2926.14
2856.70
1716.72
1600.99
1516.11
1444.75
1373.38
1280.79
1244.14
1201.70
1053.18
817.85
767.70
4000
3500
3000
2500
2000
1750
1500
1250
1000
750
Wavenumber (cm-1)

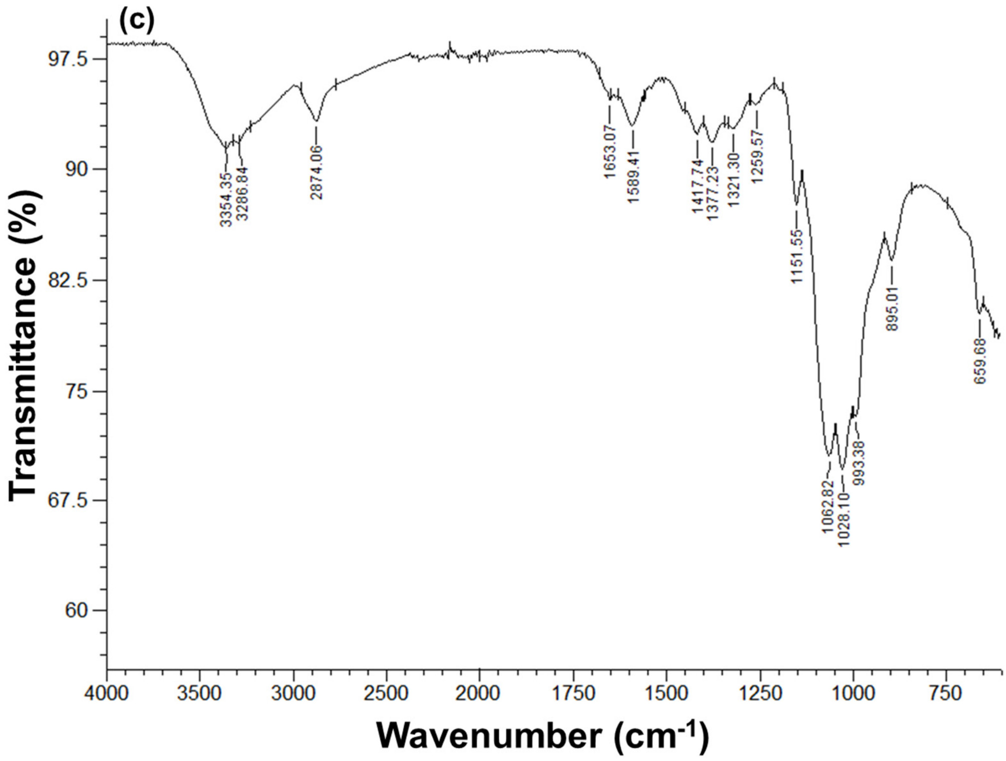
(c)
Transmittance (%)
97.5
90
82.5
75
67.5
60
3354.35
3286.84
2874.06
1653.07
1589.41
1417.74
1377.23
1321.30
1259.57
1151.55
895.01
659.68
1062.82
1028.10
993.38
4000
3500
3000
2500
2000
1750
1500
1250
1000
750
Wavenumber (cm-1)

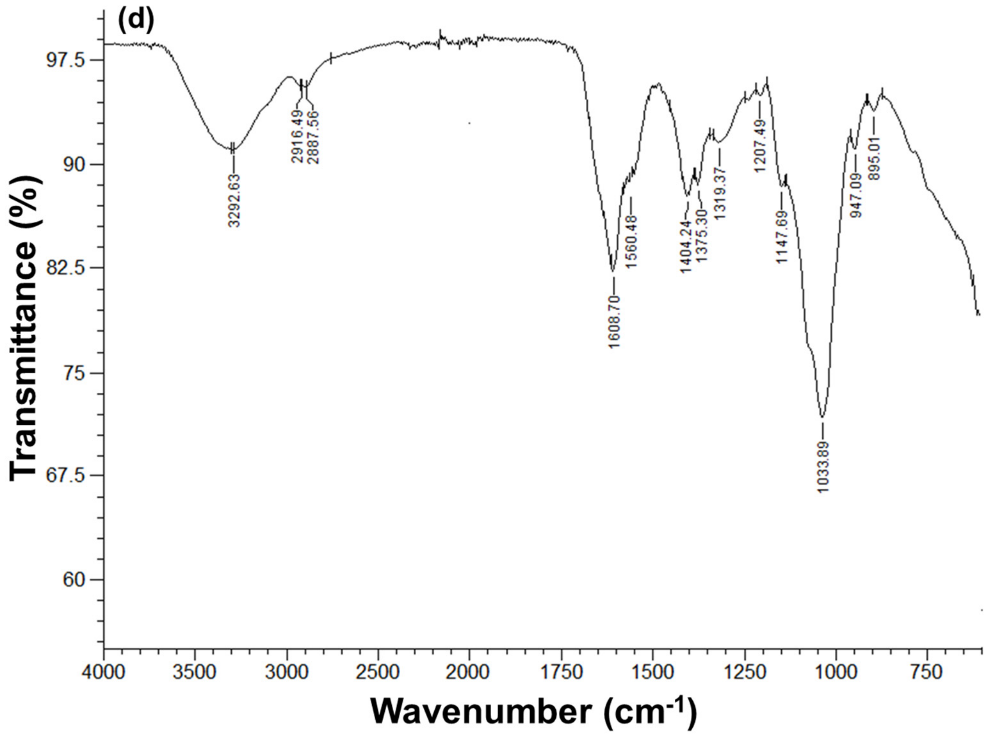
(d)
Transmittance (%)
97.5
90
82.5
75
67.5
60
3292.63
2916.49
2887.56
1608.70
1560.48
1404.24
1375.30
1319.37
1207.49
1147.69
947.09
895.01
1033.89
4000
3500
3000
2500
2000
1750
1500
1250
1000
750
Wavenumber (cm-1)

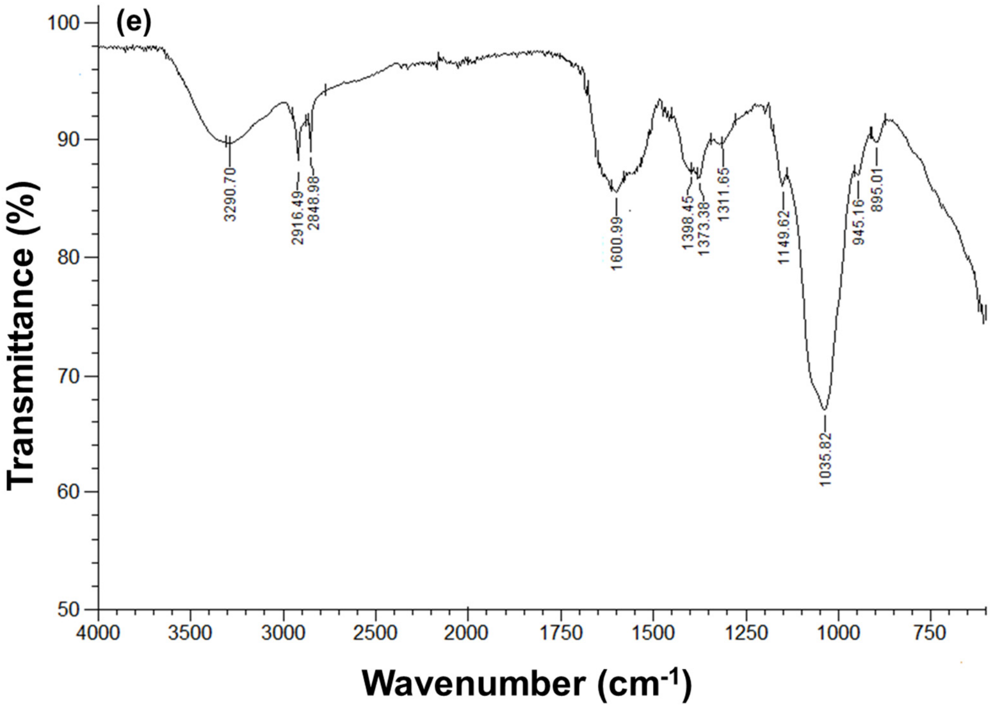

Figure S6. ATR-FTIR spectra of: (**a**) extract of MO; (**b**) extract of HP; (**c**) Ch; (**d**) HA and (**e**) PEC Ch/HA.

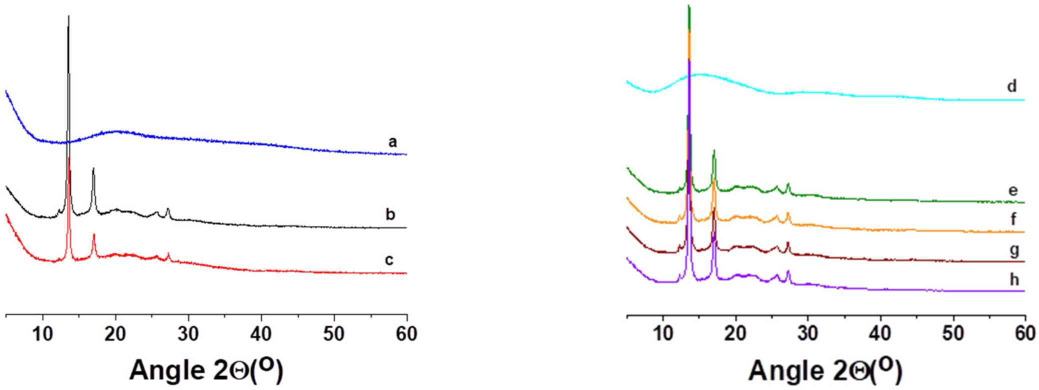

Figure S7. XRD patterns of: (**a**) MO extract; (**b**) PHB mat; (**c**) MO-*in*-PHB mat (10 wt.% MO); (**d**) HP extract; (**e**) Ch/HA-*coat*-PHB mat; (**f**) Ch/HA-*coat*-(MO-*in*-PHB) mat (10 wt.% MO); (**g**) (Ch,HP)/HA-*coat*-PHB mat (20 wt.% HP) and (**h**) (Ch,HP)/HA-*coat*-(MO-*in*-PHB) mat (10 wt.% MO, 20 wt.% HP).

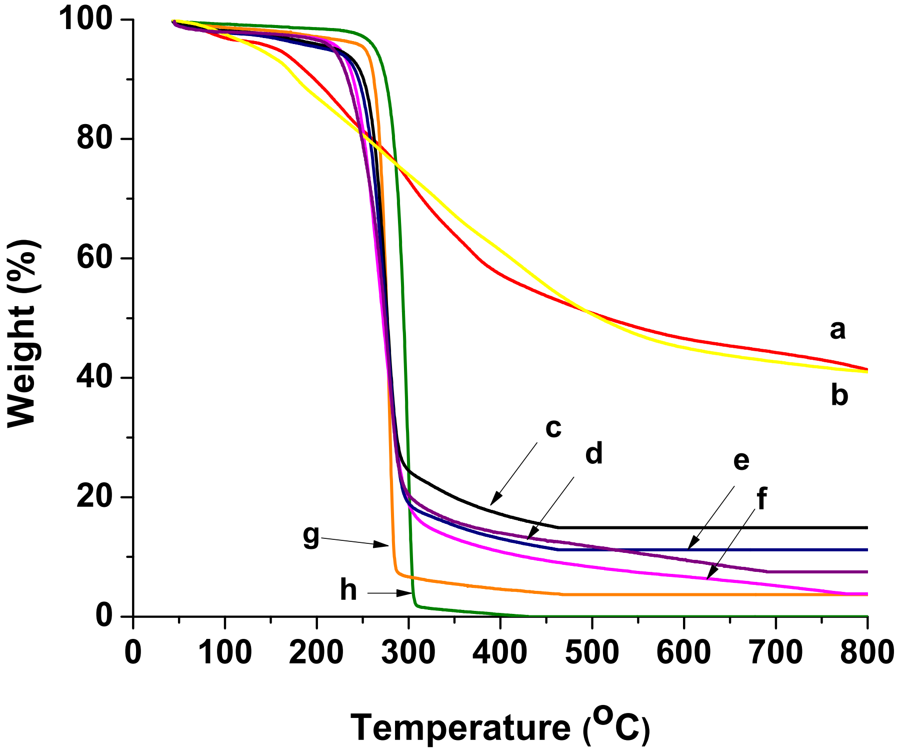

Figure S8. TGA thermograms of: (**a**) extract of MO; (**b**) extract of HP; (**c**) (Ch,HP)/HA-*coat*-(MO-*in*-PHB) mat (10 wt.% MO, 20 wt.% HP); (**d**) Ch/HA-*coat*-(MO-*in*-PHB) mat (10 wt.% MO); (**e**) (Ch,HP)/HA-*coat*-PHB mat (20 wt.% HP); (**f**) Ch/HA-*coat*-PHB mat; (**g**) MO-*in*-PHB mat (10 wt.% MO) and (**h**) PHB mat.

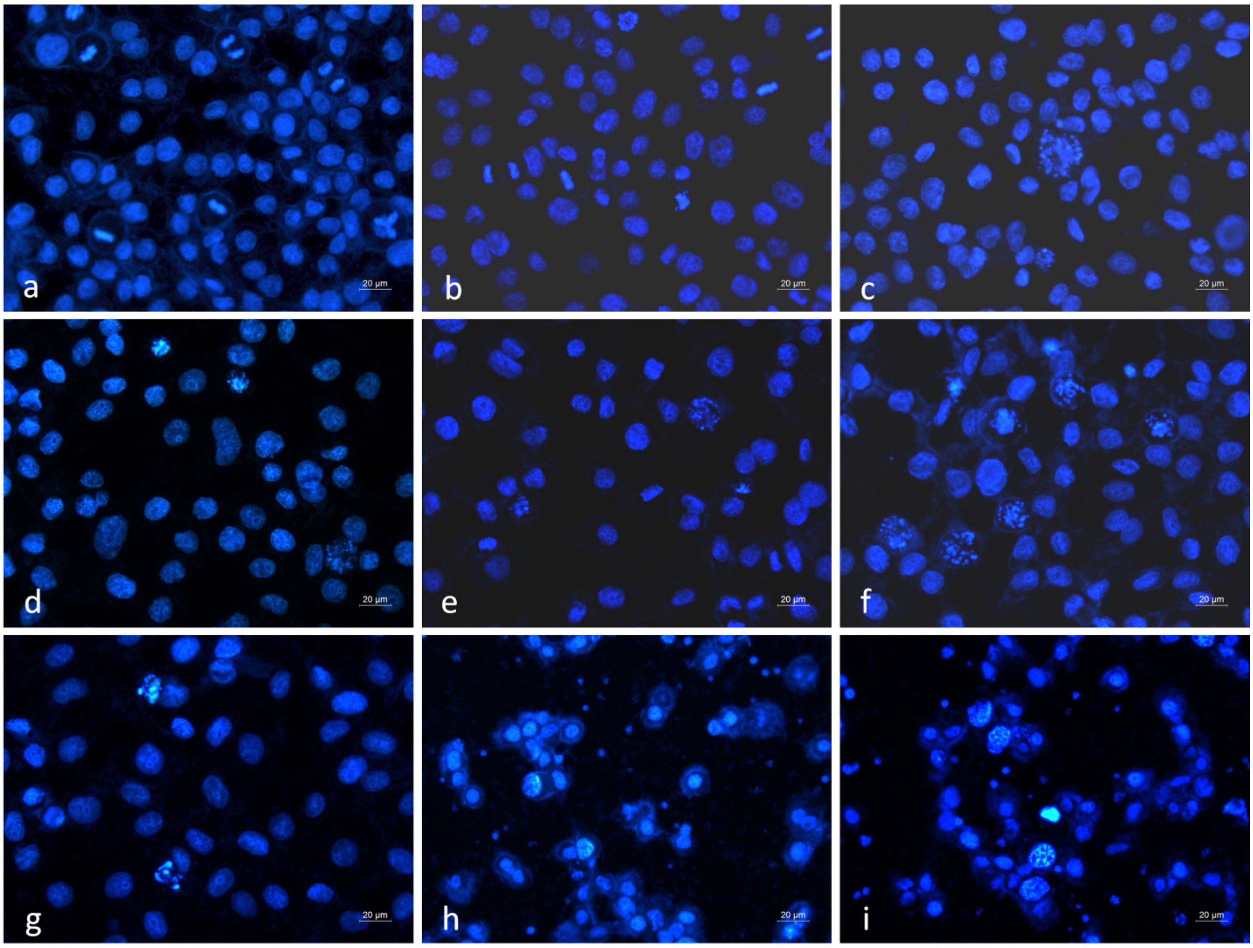

Figure S9. Fluorescence images of HeLa cancer cells stained with DAPI after 24 h of incubation with: (**a**) HeLa cells (control); (**b**) PHB mat; (**c**) Ch/HA-*coat*-PHB mat; (**d**) MO-*in*-PHB mat; (**e**) (Ch,HP)/HA-*coat*-PHB mat; (**f**) (Ch,HP)/HA-*coat*-(MO-*in*-PHB) mat; (**g**) solution of MO extract; (**h**) solution of HP extract and (**i**) solution of a mixture of MO and HP extracts (1:2 w/w). All samples containing MO and HP were investigated at a concentration of MO and HP - 130 µg/mL and 260 µg/mL, respectively.

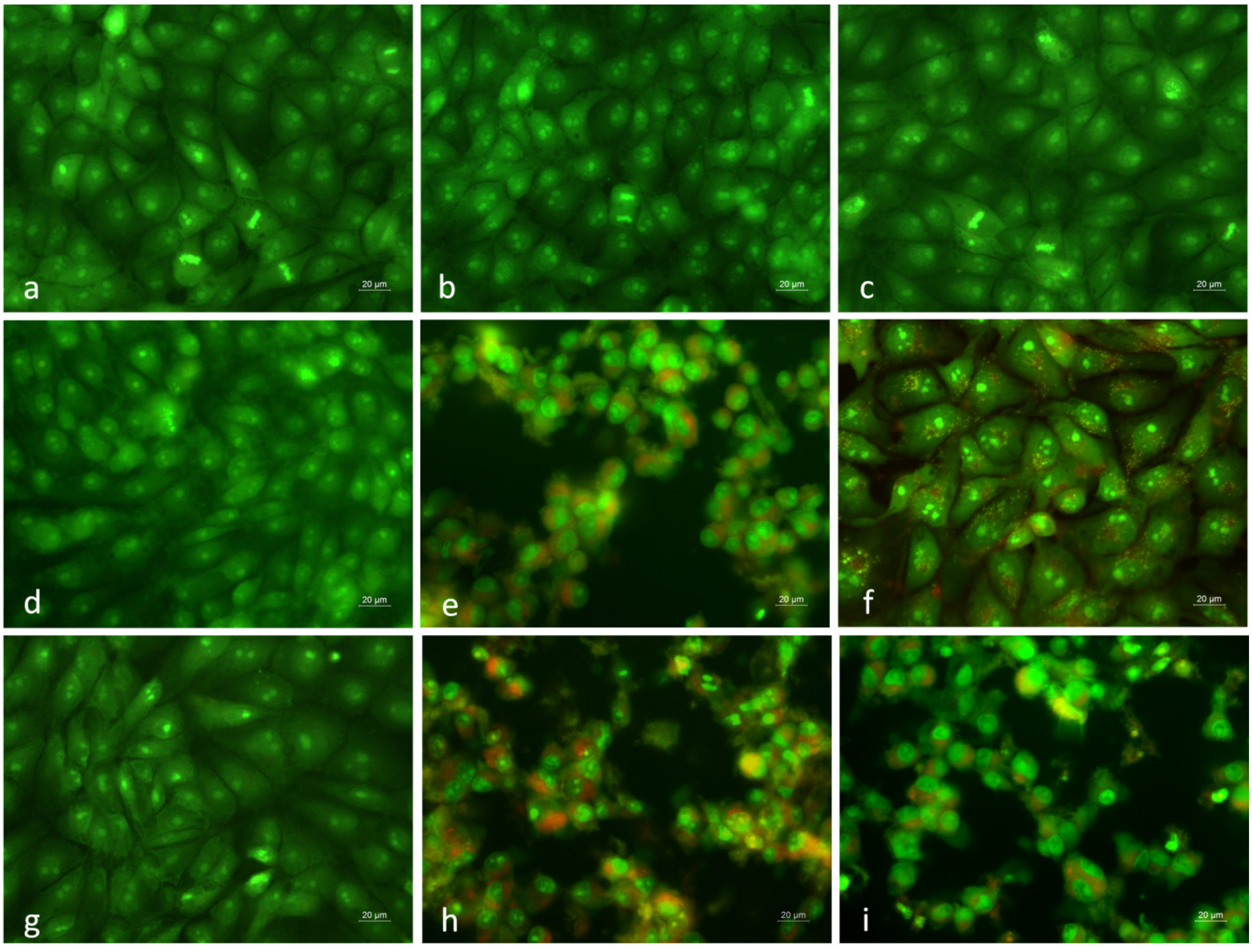

Figure S10. Fluorescence images of BALB/3T3 cells stained with AO/EtBr after 24 h of incubation with: (**a**) BALB/3T3 cells (control); (**b**) PHB mat; (**c**) Ch/HA-*coat*-PHB mat; (**d**) MO-*in*-PHB mat; (**e**) (Ch,HP)/HA-*coat*-PHB mat; (**f**) (Ch,HP)/HA-*coat*-(MO-*in*-PHB) mat; (**g**) solution of MO extract; (**h**) solution of HP extract and (**i**) solution of a mixture of MO and HP extracts (1:2 w/w). All samples containing MO and HP were investigated at a concentration of MO and HP - 130 µg/mL and 260 µg/mL, respectively.

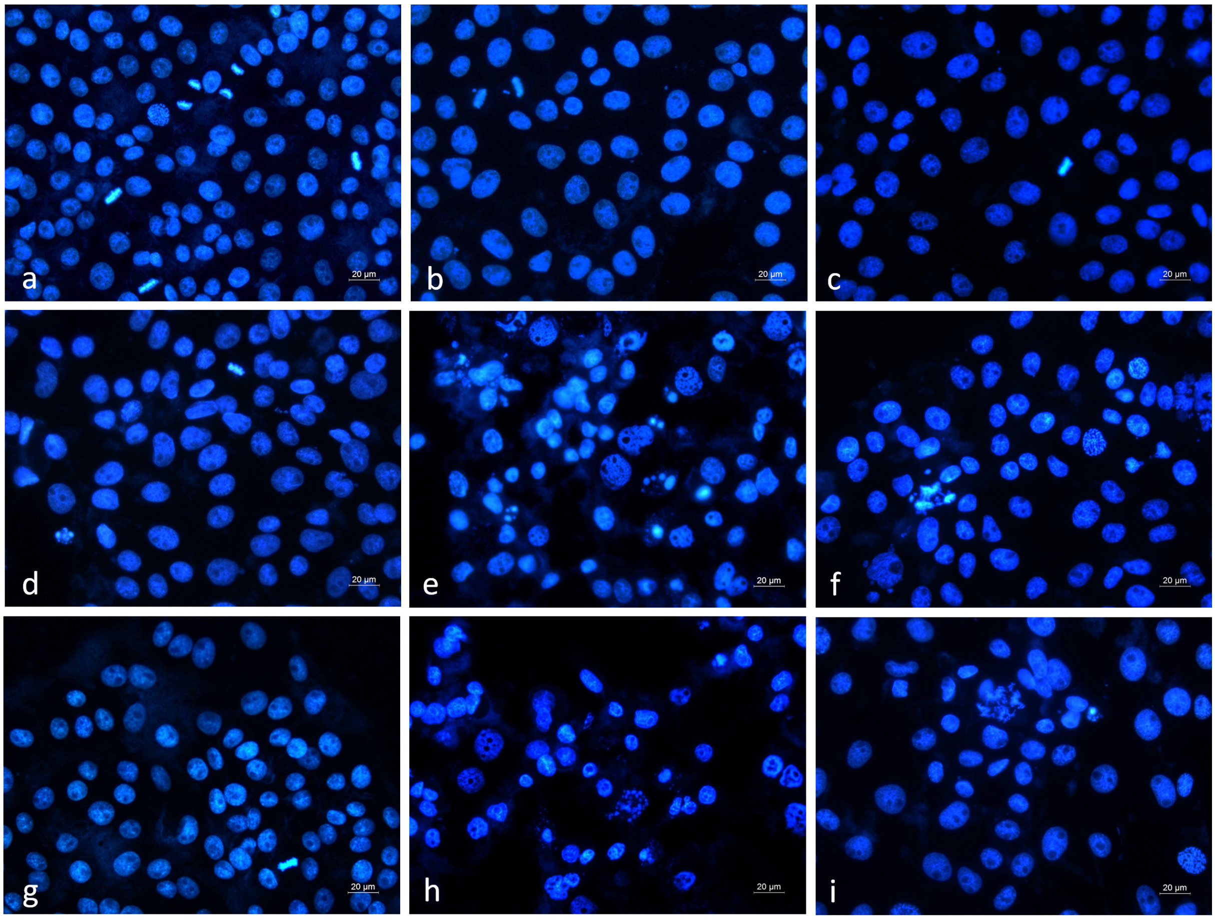

Figure S11. Fluorescence images of BALB/3T3 cells stained with DAPI after 24 h of incubation with: (**a**) BALB/3T3 cells (control); (**b**) PHB mat; (**c**) Ch/HA-*coat*-PHB mat; (**d**) MO-*in*-PHB mat; (**e**) (Ch,HP)/HA-*coat*-PHB mat; (**f**) (Ch,HP)/HA-*coat*-(MO-*in*-PHB) mat; (**g**) solution of MO extract; (**h**) solution of HP extract and (**i**) solution of a mixture of MO and HP extracts (1:2 w/w). All samples containing MO and HP were investigated at a concentration of MO and HP - 130 µg/mL and 260 µg/mL, respectively.
